# Waterlogging stress disarms nicotine‐mediated defense to herbivores through the bHLH1‐ERF1 transcriptional switch

**DOI:** 10.1111/tpj.71192

**Published:** 2026-11-09

**Authors:** Yu‐Die Xiong, Wen‐Hao Han, Jun‐Xia Wang, Jia‐Jing Wang, Chan Zhao, Yu‐Jie Chi, Yin‐Quan Liu, Shu‐Sheng Liu, Xiao‐Wei Wang

**Affiliations:** ^1^ Ministry of Agriculture and Rural Affairs Key Laboratory of Molecular Biology of Crop Pathogens and Insect Pests, Zhejiang Key Laboratory of Biology and Ecological Regulation of Crop Pathogens and Insects, Institute of Insect Sciences Zhejiang University Hangzhou 310058 China; ^2^ Rural Energy and Environment Agency Ministry of Agriculture and Rural Affairs Beijing 100125 China; ^3^ State Key Laboratory for Quality and Safety of Agro‐Products, Institute of Plant Virology Ningbo University Ningbo 315211 China

**Keywords:** waterlogging, plant defense, nicotine, transcription factor, ERF, bHLH

## Abstract

Global climate change has intensified the frequency of flooding events, which not only threaten crop productivity but also drastically alters herbivore population dynamics, often leading to more severe pest outbreaks. However, the molecular mechanisms by which waterlogging reprograms plant insect resistance remain elusive. Here we show that waterlogging stress impairs the resistance of *Nicotiana benthamiana* to herbivores by reducing nicotine accumulation through downregulation of key biosynthetic genes, *N*‐methyltransferase (PMT), and phosphoribosyltransferase (QPRT). We further reveal that this process is precisely regulated by the JA‐mediated bHLH1‐ERF1 module. Normally, this module acts as a positive regulator of nicotine biosynthesis. Under waterlogging, impaired JA synthesis in roots leads to downregulation of *NbbHLH1*, thereby suppressing *NbERF1* expression. Consequently, the downregulation of both NbERF1 and NbbHLH1 directly switches off the transcription of *NbPMT* and *NbQPRT*, leading to reduced nicotine accumulation and compromised plant resistance. Critically, the bHLH1‐ERF1 module also negatively regulates waterlogging tolerance, thereby acting as a transcriptional switch that disarms defense to prioritize survival. These findings reveal the molecular mechanism governing plant trade‐offs under combined stresses.

## SUMMARY

Waterlogging stress reduces root jasmonic acid levels, downregulating the *NbbHLH1*‐*NbERF1* signaling module. This suppression diminishes the expression of key biosynthetic genes (*NbPMT* and *NbQPRT*), leading to decreased nicotine accumulation in the leaves and increased susceptibility to chewing herbivores.

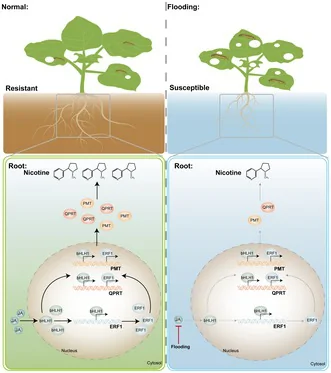

Global climate change‐induced flooding often triggers severe pest outbreaks by compromising plant defenses through poorly understood molecular mechanisms. This study identifies the JA‐mediated bHLH1‐ERF1 module as a critical molecular switch that prioritizes waterlogging tolerance over nicotine‐based herbivore resistance, providing a strategic framework for developing resilient crops capable of enduring combined environmental challenges.

## INTRODUCTION

With global warming, the frequency of extreme weather events such as floods is increasing worldwide (Bolan et al., 2024). According to the United Nations, the number of floods in the world had doubled over the past 20 years (Tellman et al., 2021). Approximately 10% of agricultural fields are severely affected by waterlogging annually (Kaur et al., 2020). Waterlogging stress induced by flooding events disrupts gas exchange in plant tissues, which consequently impairs mitochondrial respiration and disrupts normal biochemical processes (Jia et al., 2021; Renziehausen et al., 2024; Yeung et al., 2018). Beyond these direct physiological disruptions, waterlogging can also alter plant defense mechanisms (Garcia et al., 2024; Martínez‐Arias et al., 2022). Herbivore outbreaks are often observed after floods. For instance, large populations of *Ips typographus*, *Pityogenes chalcographus*, and *Plagiodera versicolora* frequently appear following waterlogging events (Dávalos & Blossey, 2010; Nakamura et al., 2005; Schroeder & Lindelöw, 2012). These observations raise the question of how waterlogging affects plant resistance.

Being sessile organisms constantly exposed to various biotic stresses, plants have evolved diverse defense strategies, which can be broadly categorized into physical and chemical defense (Bouwmeester et al., 2019; Erb & Reymond, 2019; Fernandez de Bobadilla et al., 2022; Schuurink & Tissier, 2020). Physical defenses involve structural adaptations, such as reinforced cell walls, trichomes and lignin deposition, which help protect the plant from herbivore damage (Han et al., 2024; Ji et al., 2024; Wang et al., 2025; Xu et al., 2024; Zocca et al., 2025). In contrast, chemical defenses refer to the production of various chemical compounds that enable plants to resist or slow the attack of pests, which include protein inhibitors, secondary metabolites and volatile organic compounds (Ali et al., 2019; Mithofer & Boland, 2012; Wang et al., 2025). While it is well‐established that plants utilize both physical and chemical defenses to fend off herbivores, the effects of waterlogging on these defense mechanisms remain largely unexplored. Previous study shows that waterlogging can reduce insecticidal protein content in cotton, thus impairing its resistance to cotton bollworm (Zhou et al., 2015). Similarly, waterlogging has been found to lower jasmonic acid (JA) levels, thereby weakening the resistance of *Arabidopsis* to *Pieris rapae* (Lee et al., 2020). In rice, waterlogging reduces the content of key metabolites involved in plant defense (Lu et al., 2015). In contrast, a recent study in maize reported that waterlogging induces salicylic acid (SA) accumulation and flavonoid biosynthesis, thereby enhancing plant resistance to herbivores (Gorman et al., 2025). However, how waterlogging affects plant resistance to herbivores and the underlying molecular mechanisms remain poorly understood.

Nicotine is one of the most important chemical compounds in Solanaceae plants (Steppuhn et al., 2004; Steppuhn & Baldwin, 2007). Nicotine not only exerts direct neurotoxic effects, causing insect death by disrupting their nervous system, but also reduces insect damage to plants through a feeding deterrent mechanism (Steppuhn et al., 2004). Previous studies showed nicotine can help plants resist a variety of herbivores, such as *Manduca sexta*, *Myzus persicae* and *Anthonomus grandis* (Ewing & Moreland, 1942; Gaines, 1942; Gaines, 1944; Zhao et al., 2016). Mechanical damage caused by insect feeding or JA can induce nicotine formation (Baldwin, 1998; Steppuhn et al., 2004). Nicotine consists of a pyrrolidine and a pyridine ring, synthesized through distinct amino acid pathways (Wagner et al., 1986). The pyrrolidine ring is derived from arginine and ornithine via enzymes such as arginine decarboxylase (ADC) and putrescine *N*‐methyltransferase (PMT), while the pyridine ring originates from aspartic acid and involves enzymes like quinolinate phosphoribosyl transferase (QPRT) (Shoji et al., 2024). Among these, PMT and QPRT are rate‐limiting enzymes and critical regulators of nicotine biosynthesis (Feth et al., 1986; Saunders & Bush, 1979; Wagner & Wagner, 1985). However, the regulatory mechanisms controlling nicotine accumulation and the expression of its key biosynthetic genes under abiotic stress conditions remain to be elucidated.

Cotton bollworm (*Helicoverpa armigera*), fall armyworm (*Spodoptera frugiperda*), and tobacco caterpillar (*Spodoptera litura*) are major pests of Solanaceae crops, causing substantial agricultural losses (Riaz et al., 2021; Wang et al., 2020). Previous studies indicate that populations of *H. armigera* and *S. frugiperda* exhibit significant correlations with waterlogging stress (Silvain & Tiahing, 1985; Xie et al., 2018). For example, analysis of *S. frugiperda* populations in French Guiana over 75 weeks revealed increased larval abundance during rainy seasons (Silva‐Sanzana et al., 2020). Similarly, in Nanyang, China, *H. armigera* populations surged after waterlogging events, leading to its designation as a key prevention target in flood‐affected regions (Xie et al., 2018). *Nicotiana benthamiana*, a model plant in the Solanaceae family, is widely used in plant‐biotic interactions research (Chen et al., 2024). We also found that *N. benthamiana*, characterized by its multifaceted anti‐herbivore traits, including physical barriers (e.g. lignin) and specialized metabolites (e.g. acyl sugars), can be used as a dead‐end trap to control whitefly and aphids in the field (Han et al., 2024; Ji et al., 2024; Wang et al., 2025). During our field experiments, we observed that in waterlogged fields, chewing insects caused more severe damage to *N. benthamiana* plants, suggesting that waterlogging compromises plant resistance to chewing herbivores. This phenomenon presents a valuable opportunity to investigate the molecular mechanisms underlying plant trade‐offs between waterlogging tolerance and herbivore defense.

We investigated how waterlogging stress affects herbivore resistance in *N. benthamiana* and found that it significantly reduces nicotine content by downregulating key biosynthetic genes, including the rate‐limiting enzymes *NbPMT* and *NbQPRT*. Mechanistic analysis revealed that these genes are co‐regulated by a transcriptional module consisting of *NbERF1* and *NbbHLH1*, where NbbHLH1 directly activates *NbERF1* expression and together they promote nicotine biosynthesis. Waterlogging suppresses this pathway by downregulating *NbbHLH1*, which in turn reduces *NbERF1* expression levels. These results elucidate a novel mechanism linking waterlogging stress to plant defense modulation and highlight how environmental stressors reshape plant–herbivore interactions.

## RESULTS

### Waterlogging inhibits plant defense against several herbivores

To assess the impact of waterlogging on plant resistance, the roots of *N. benthamiana* plants were submerged in water in a tray for 7 days (Figure 1a). Compared to the control plant, the waterlogging treatment resulted in characteristic stress phenotypes: reduced leaf size and impaired growth (Figure 1b). After 7 days of waterlogging, second‐instar larvae of *Helicoverpa armigera*, *Spodoptera litura*, and *Spodoptera frugiperda* that had been starved for 24 h were placed on these plants. Their weights were recorded after 48 h. Notably, all three herbivore species gained significantly more weight on waterlogging treatment plants than on control plants (Figure 1c–h). These findings suggest that waterlogging reduces the plant's ability to defend against herbivores.

**Figure 1:**
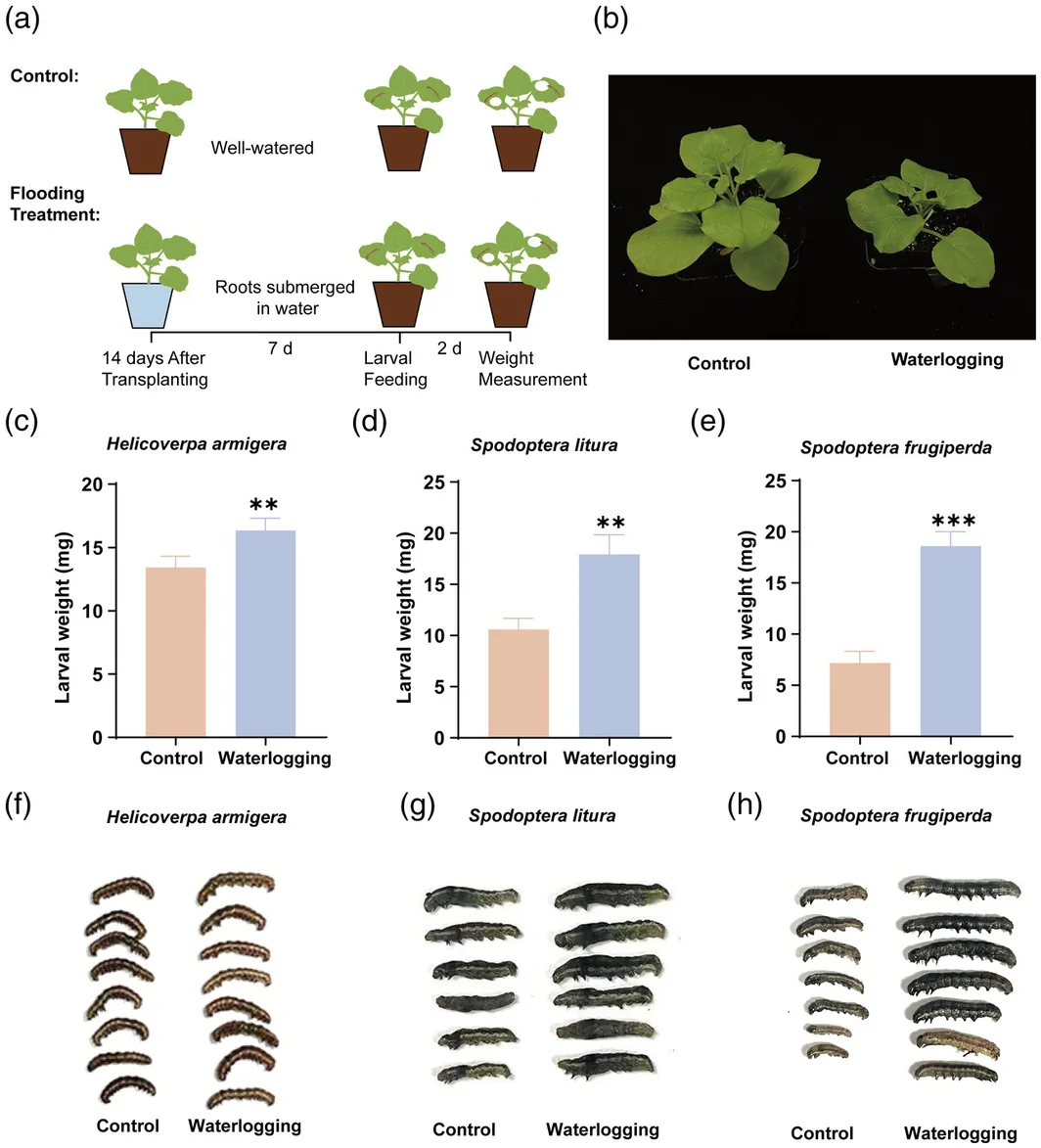
Herbivores perform better on waterlogged *N. benthamiana* compared to the control. (a) Schematic diagram of the experimental design. *N. benthamiana* plants were maintained under well‐watered (control) or waterlogged conditions for 7 days. On day 7, plants were inoculated with herbivores. After 2‐day feeding, larval weight was measured. (b) Photographic records of plant morphology under control and flooded treatments. (c–h) Larval weight and corresponding morphological photo under different treatments: (c) *Helicoverpa armigera*, (d) *Spodoptera litura*, (e) *Spodoptera frugiperda*. Each species is shown under control (left panels) and waterlogging treatment (right panels), with representative larvae morphology as shown in (f) *Helicoverpa armigera*, (g) *Spodoptera litura*, and (h) *Spodoptera frugiperda*. Values represent mean ± SE, *n* = 14 biological replicates for (c), *n* = 8–10 biological replicates for (d), *n* = 7 biological replicates for (e). Student's *t*‐test (two‐tailed) was used for significance analysis. *P < 0.05, **P < 0.01, ***P < 0.001.

### Waterlogging does not alter plant hormone level, trichome density, and lignin content

To examine how waterlogging affects plant resistance to herbivores, we measured the levels of key plant hormones involved in defense against herbivores. Jasmonic acid (JA) and salicylic acid (SA) are essential for plant responses to biotic stress (Erb et al., 2012). Our results showed no significant differences in JA and SA levels in leaves between the control and waterlogging treatment (Figure S1a,b). This indicates that waterlogging affects plant resistance to *H. armigera* independently of JA and SA levels in the leaves.

Previous studies have shown that trichomes play an important role in *N. benthamiana* defense against herbivores (Han et al., 2024; Wang et al., 2025). However, no significant differences were observed in trichome numbers between 7‐day waterlogged and control plants (Figure S1c). Lignin, a phenolic polymer predominantly deposited in the secondary cell walls, is also critical in plant‐herbivore interactions (Ji et al., 2024). However, waterlogging did not induce any significant changes in lignin content in *N. benthamiana* (Figure S1d).

### Waterlogging inhibits the biosynthesis of nicotine

Nicotine is a major chemical weapon in *Nicotiana* (Shoji et al., 2024). We measured nicotine levels in *N. benthamiana* under control and waterlogging conditions. The results showed that nicotine content in waterlogged plant leaves was significantly lower than in control plant leaves (Figure 2a). To explore the underlying mechanism of nicotine reduction, we examined the expression of genes involved in nicotine biosynthesis (Figure 2b). Given that nicotine is root‐synthesized and xylem‐transported to leaves for anti‐herbivore defense, we detected nicotine biosynthesis genes in the roots (Dawson, 1941). Notably, the expression levels of aspartate oxidase (AOX), quinolinate synthetase (QS), quinolinate phosphoribosyltransferase (QPRT), ornithine decarboxylase (ODC), and putrescine *N*‐methyltransferase (PMT), which encode key enzymes responsible for the biosynthesis of the pyrrolidine and pyridine rings, respectively, were significantly downregulated in waterlogged *N. benthamiana* (Figure 2b,c). *NbQPRT* and *NbPMT* are known to play critical roles in nicotine biosynthesis (Dewey & Xie, 2013). Silencing of *NbPMT* and *NbQPRT* in *N. benthamiana* resulted in a significant decrease in nicotine levels and a corresponding weakening of resistance to *H. armigera* (Figure 2d,e; Figure S2). This is consistent with previous studies demonstrating that PMT and QPRT are required for nicotine biosynthesis and that nicotine plays a key role in plant defense against herbivores (Feth et al., 1986; Steppuhn et al., 2004; Wagner & Wagner, 1985). In summary, our study highlights the detrimental effects of waterlogging on nicotine biosynthesis and plant defense to insects and identifies the downregulation of *NbPMT* and *NbQPRT* as critical factors in this process.

**Figure 2:**
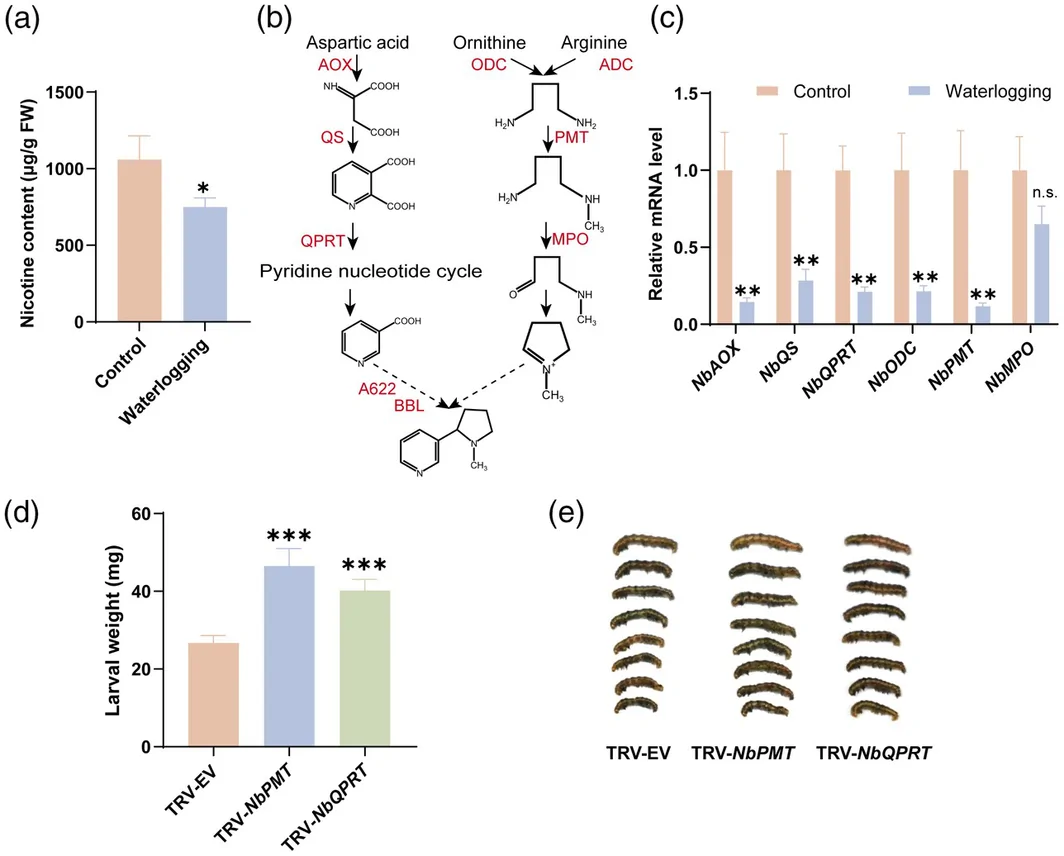
Nicotine content and the expression levels of its biosynthetic genes were significantly downregulated in waterlogged *N. benthamiana* plants. (a) Nicotine content in *N. benthamiana* leaves from well‐watered and 7‐day flooded plants. (b) Proposed nicotine biosynthetic pathway. Dashed arrows represent undefined or multiple steps. A622, isoflavone reductase‐like protein A622; ADC, arginine decarboxylase; AOX, aspartate oxidase; BBL, berberine bridge enzyme‐like protein; MPO, *N*‐methylputrescine oxidase; ODC, ornithine decarboxylase; PMT, putrescine *N*‐methyltransferase; QPRT, quinolinate phosphoribosyltransferase; QS, quinolinate synthetase. (c) Expression levels of nicotine biosynthetic genes in well‐watered (control) and waterlogging‐treated *N. benthamiana* plants. (d) Weight of *H. armigera* feeding on TRV‐EV (control), TRV‐*NbPMT*, and TRV‐*NbQPRT N. benthamiana* plants. (e) Representative images of *H. armigera* after feeding on TRV‐EV (control), TRV‐*NbPMT*, and TRV‐*NbQPRT N. benthamiana* plants. Values represent mean ± SE, *n* = 3 biological replicates for (a) (each replicate consisted of a pool of two plants); *n* = 11–12 biological replicates for (c); *n* = 8–16 biological replicates for (d). Student's *t*‐test (two‐tailed) was used for significance analysis. *P < 0.05, **P < 0.01, ***P < 0.001.

### *NbERF1* binds to the promoters of *NbPMT* and *NbQPRT* to activate their transcription

To identify transcription factors regulating *NbPMT* and *NbQPRT* expression, we conducted yeast one‐hybrid (Y1H) screening using the promoter of *NbPMT* and *NbQPRT* with a cDNA library derived from *N. benthamiana*. Yeast cells co‐transformed with constructs containing *NbERF1* and the promoters of *NbPMT* and *NbQPRT* exhibited robust growth on selective medium, whereas those transformed with the empty vector (EV) did not grow, demonstrating that NbERF1 interacts with the promoters of *NbPMT* and *NbQPRT* (Figure 3a,b). Through PlantPAN analysis, we predicted multiple ERF‐binding sites in the promoters of both *NbPMT* and *NbQPRT* (Figure 3c). Fluorescence confocal experiment revealed that NbERF1 is localized in the nucleus, a defining characteristic of transcription factors (Figure S3). Conserved domain analysis revealed that NbERF1 contains a highly conserved AP2/ERF DNA‐binding domain (Figure S4). Given that NtERF189 and NtERF199 are known to regulate nicotine biosynthesis in *Nicotiana tabacum* (Shoji & Hashimoto, 2011), we performed a multiple sequence alignment analysis. The results showed that NbERF1 shares high amino acid identity with NtERF189 (71.12%) and NtERF199 (69.04%) (Figure S4). Moreover, NbERF1 clusters with both genes in a well‐supported clade and is therefore considered their ortholog in *N. benthamiana* (Shoji & Hashimoto, 2012). Next, we used GUS reporter assays to test the transactivation activity of NbERF1 on *NbPMT* and *NbQPRT* promoters. In plants overexpressing *NbERF1* (*NbERF1*‐OE), the expression of *NbPMTpro*::GUS and *NbQPRTpro*::GUS was significantly higher than in plants carrying the control (1305‐GFP), indicating that *NbERF1* positively regulates the transcription of these genes (Figure 3d–g). Coexpression of *NbERF1* with the LUC reporters resulted in higher LUC activity compared to the control, suggesting that NbERF1 activated these promoters (Figure S5). Furthermore, transient expression of *NbERF1* significantly elevated the transcript levels of both *NbPMT* and *NbQPRT* (Figure 3h). To further validate binding specificity, we generated an NbERF1 mutant lacking the AP2/ERF DNA‐binding domain (NbERF1‐ΔDB). This mutant failed to interact with either promoter in Y1H assays and did not activate their transcription in GUS assays, confirming that NbERF1 directly binds to and transcriptionally activates *NbPMT* and *NbQPRT* (Figure S6). To test whether a single conserved cis‐element is responsible for NbERF1 binding, we introduced mutations into the predicted GCC‐box‐like motifs in both promoters. These motifs were selected because they match the ERF189 consensus (A/C)GC(A/C)(A/C)NCC, which was previously shown to be important for ERF binding (Shoji & Hashimoto, 2012). However, while Y1H assays showed that NbERF1 retained binding to the mutated promoters, GUS reporter assays revealed that the transactivation activity of NbERF1 on the mutated promoters was markedly reduced (Figure S7), suggesting that multiple ERF‐binding sites exist in the *NbPMT* and *NbQPRT* promoters and may function redundantly.

**Figure 3:**
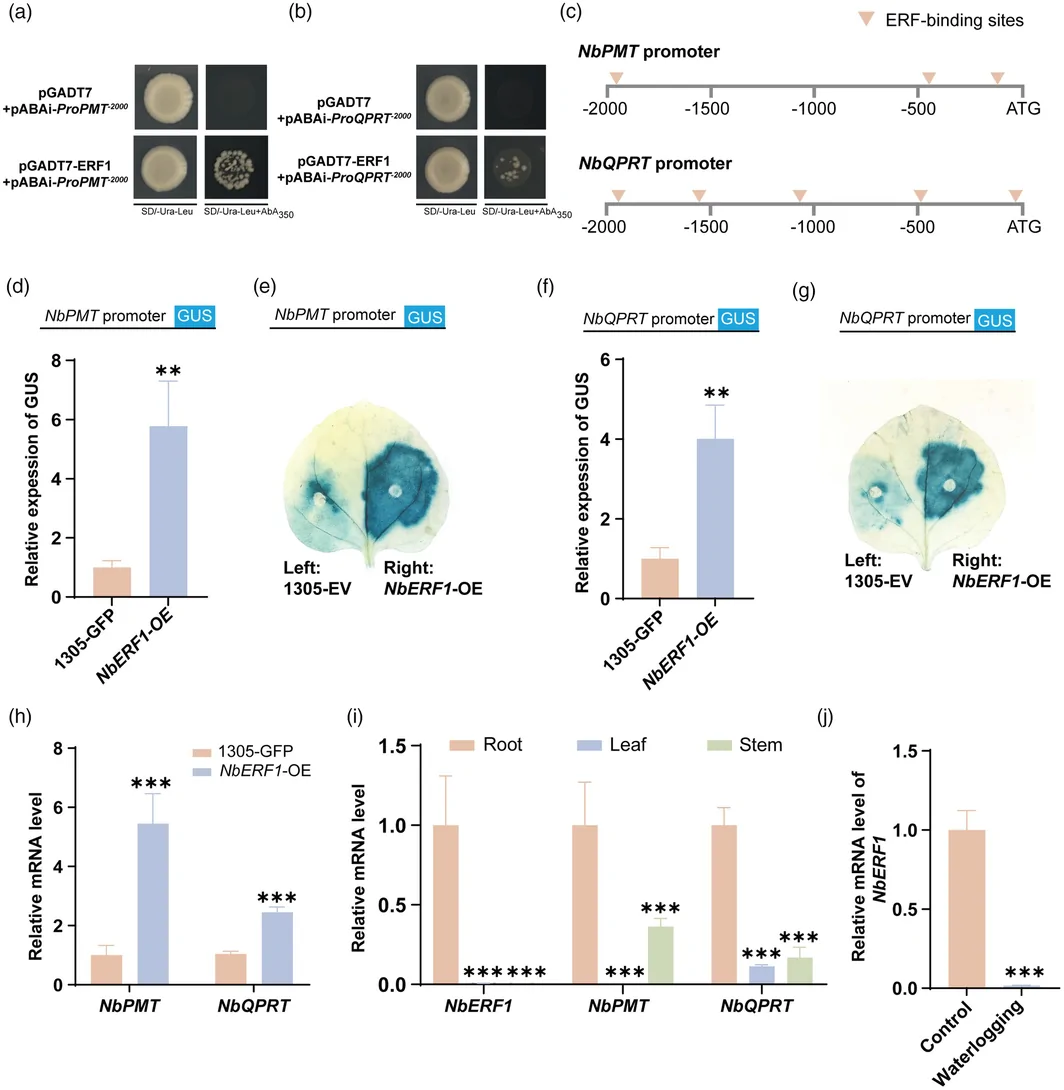
NbERF1 binds to the promoters of *NbPMT* and *NbQPRT* to activate their transcription. (a, b) Y1H assay showing the interaction of NbERF1 with the promoters of *NbPMT* and *NbQPRT*. Yeast co‐transformed with pGADT7‐NbERF1 and pAbAi‐*NbPMTpro* (a) or pAbAi‐*NbQPRTpro* (b) grew on SD/Leu medium with or without AbA (350 ng/mL). Empty pGADT7 served as a control. (c) Sequence analysis of *NbPMT* and *NbQPRT* promoters with triangles marking ERF‐binding sites. (d, e) Validation of the interaction between NbERF1 and the *NbPMT* promoter by GUS reporter gene expression (d) and histochemical staining (e). (f, g) Validation of the interaction between NbERF1 and the *NbQPRT* promoter by GUS reporter gene expression (f) and histochemical staining (g). (h) Expression levels of *NbPMT* and *NbQPRT* in 1305‐GFP (control) and *NbERF1*‐OE *N. benthamiana* plants. (i) Expression levels of *NbERF1*, *NbPMT*, and *NbQPRT* in different plant tissues. (j) Expression levels of *NbERF1* in control and flooded *N. benthamiana* plants. Values represent mean ± SE, *n* = 6 biological replicates for (d, f); *n* = 6–8 biological replicates for (h); *n* = 11 for (i); *n* = 12 biological replicates for (j). Student's *t*‐test (two‐tailed) was used for significance analysis. **P < 0.01, ***P < 0.001.

Further, we measured *NbERF1* transcript levels in different *N. benthamiana* organs using RT‐qPCR and found that *NbERF1* is specifically expressed in the roots, consistent with the expression patterns of *NbPMT* and *NbQPRT* (Figure 3i). Significantly, under waterlogging stress, *NbERF1* expression was concomitantly suppressed with its target genes in roots (Figure 3j; Figure 2c). In contrast, no significant changes in the expression levels of NbERF1 were observed in stems or leaves under waterlogging (Figure S8), indicating tissue‐specific transcriptional repression. This coordinated downregulation provides a mechanistic explanation for waterlogging‐induced repression of nicotine biosynthesis genes, wherein *NbERF1* appears to function as a key transcriptional regulator linking environmental cues to plant defense metabolism.

### *NbERF1* positively regulates plant resistance to herbivores

Having established that *NbERF1* directly activates *NbPMT* and *NbQPRT* transcription, we next examined whether *NbERF1* expression affects nicotine biosynthesis and herbivore resistance. We generated *N. benthamiana* transgenic lines either overexpressing *NbERF1* or harboring RNAi constructs for *NbERF1* silencing. RT‐qPCR analyses confirmed successful transgene RNAi lines (Lines 1 and 4) (Figure S9a). Phenotypically, no significant differences were observed between wild‐type (WT) plants and the *Nb‐ERF1*i transgenic lines in a normal environment (Figure 4a). As anticipated, the relative expression levels of key nicotine biosynthetic genes *NbPMT* and *NbQPRT* were downregulated in the *Nb‐ERF1*i lines, concomitant with an increase in *H. armigera* larval weight compared to WT (Figure 4b–e). Western blot and RT‐qPCR analyses confirmed successful transgene integration in a representative overexpression line (Figure S9b,c). p35S::*NbERF1* lines also showed no significant differences in growth compared to WT (Figure 4f). Subsequent transcriptional profiling revealed significant upregulation of most nicotine biosynthetic genes in p35S::*NbERF1*, with *NbPMT* and *NbQPRT* expression levels significantly higher than in WT plants (Figure 4g,h). And the overexpression line showed stronger resistance to *H. armigera* compared to WT (Figure 4i,j). Consistent with these results, the nicotine content decreased in *NbERF1*i transgenic lines and increased in p35S::*NbERF1* (Figure 4k). Collectively, *NbERF1* transcriptionally activates herbivore resistance by regulating the nicotine pathway.

**Figure 4:**
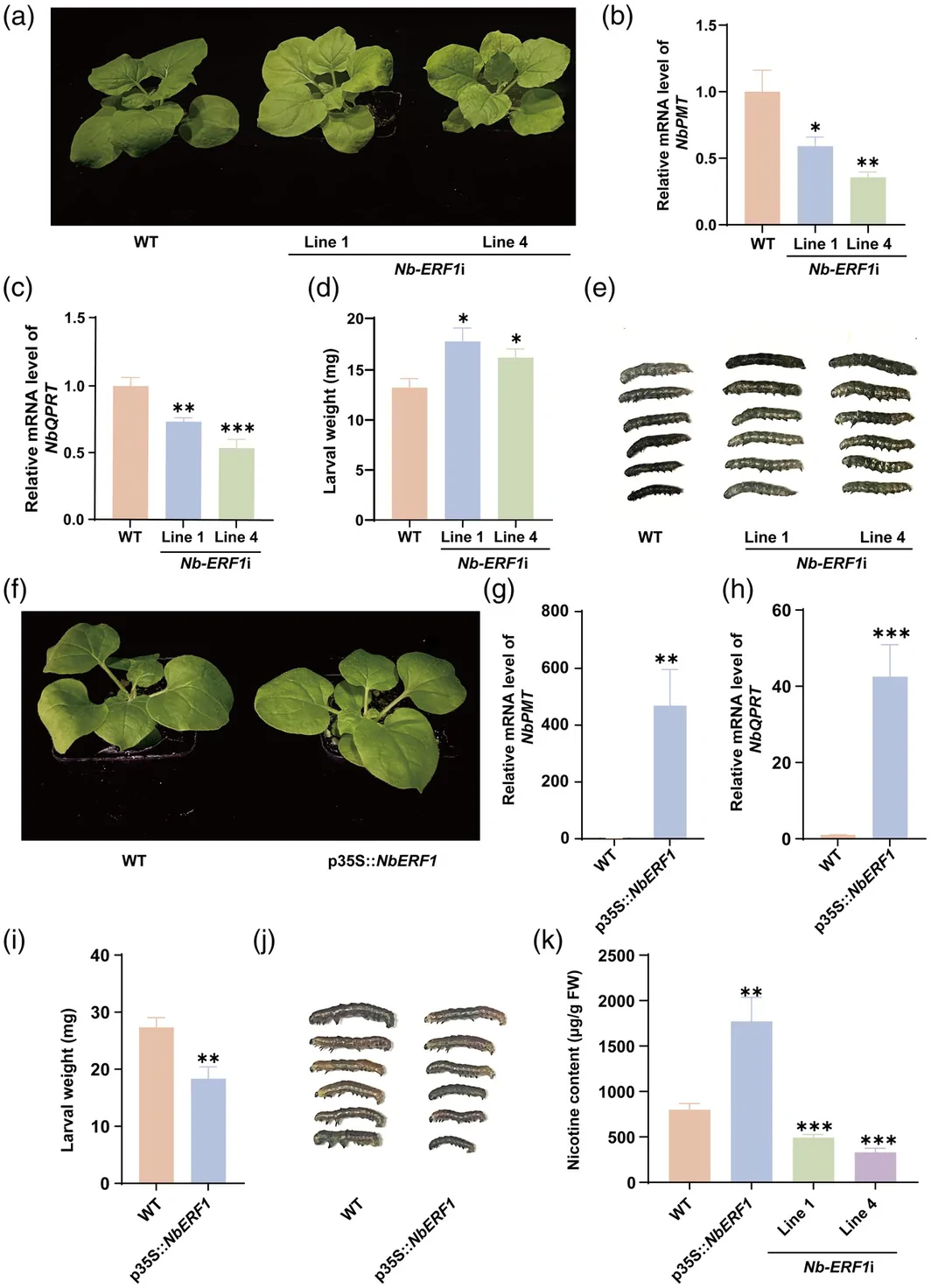
*NbERF1* positively regulates plant resistance in *N. benthamiana* plants. (a) Phenotypes of *Nb‐ERF1*i transgenic *N. benthamiana* plants. (b, c) Expression levels of *NbPMT* (b) and *NbQPRT* (c) in WT and *Nb‐ERF1*i transgenic *N. benthamiana* plants. (d) Weight of *H. armigera* feeding on WT and RNAi‐*NbERF1* transgenic *N. benthamiana* plants. (e) Representative images of *H. armigera* after feeding on WT and *Nb‐ERF1*i transgenic *N. benthamiana* plants. (f) Phenotypes of p35S::*NbERF1* transgenic *N. benthamiana* plants. (g, h) Expression levels of *NbPMT* (g) and *NbQPRT* (h) in WT and p35S::*NbERF1* transgenic *N. benthamiana* plants. (i) Weight of *H. armigera* feeding on WT and p35S::*NbERF1* transgenic *N. benthamiana* plants. (j) Representative images of *H. armigera* after feeding on WT and p35S*::NbERF1* transgenic *N. benthamiana* plants. (k) Nicotine content in WT, p35S::*NbERF1*, and *Nb‐ERF1*i transgenic *N. benthamiana* plants. Values represent mean ± SE, *n* = 6–8 biological replicates for (b, c); *n* = 10–13 biological replicates for (d); *n* = 6 for (g and h); *n* = 11 biological replicates for (i), *n* = 6 biological replicates for (k) (each replicate consisted of a pool of two plants). Student's *t*‐test (two‐tailed) was used for significance analysis. *P < 0.05, **P < 0.01, ***P < 0.001.

We also evaluated the defense ability of p35S::*NbERF1* and *Nb*‐*ERF1*i plants against herbivores under waterlogging conditions. The results showed that p35S::*NbERF1* plants exhibited stronger resistance compared with WT plants under waterlogging stress, whereas *Nb*‐*ERF1*i plants displayed the opposite phenotype (Figure S10). These findings further demonstrate that ERF contributes to insect defense under waterlogging stress.

### *NbbHLH1* governs dual transcriptional control via *NbERF1* relay and direct activation of nicotine biosynthesis genes

To dissect the upstream regulation mechanism of *NbERF1*, we analyzed its promoter via PlantPAN and found multiple E‐box motifs (HLH TF binding sites; Figure 5a). Previous studies have shown *NbbHLH1* (basic helix–loop–helix) is involved in nicotine biosynthesis and binds to E‐box motifs (Todd et al., 2010). We thus hypothesized that NbbHLH1 may directly regulate *NbERF1* expression. Overexpression of *NbbHLH1* in *N. benthamiana* H2B RFP line showed that NbbHLH1 was located in the nucleus, a feature of transcription factors (Figure S11). To confirm direct binding between NbbHLH1 and the *NbERF1* promoter, Y1H assays were conducted. Yeast cells co‐transformed with NbbHLH1 prey and the *NbERF1* promoter displayed robust growth on selective medium, while cells transformed with the EV showed no growth, indicating that NbbHLH1 specifically interacts with the NbERF1 promoter in yeast (Figure 5b). Next, we examined the activity of the *NbERF1* promoter using *NbERF1pro*::GUS reporter constructs in *NbbHLH1*‐overexpressing plants. GUS staining showed significantly higher promoter activity in *NbbHLH1*‐OE plants compared to the control, indicating that NbbHLH1 positively regulates the *NbERF1* promoter (Figure 5c,d). Transient expression assays with LUC constructs driven by the *NbERF1* promoter further confirmed this regulation (Figure S12). Notably, overexpression of *NbbHLH1* dramatically induced the expression of *NbERF1* (Figure 5e). Consistent with this, silencing of *NbbHLH1* via VIGS not only compromised resistance to herbivores but also downregulated *NbERF1* expression (Figure 5f; Figure S13). These results collectively suggest that *NbbHLH1* directly activates *NbERF1* expression to confer insect resistance. Furthermore, we found that waterlogging suppresses *NbbHLH1* expression in the roots (Figure 5g). This suggests that waterlogging may downregulate *NbERF1* through the suppression of *NbbHLH1*.

**Figure 5:**
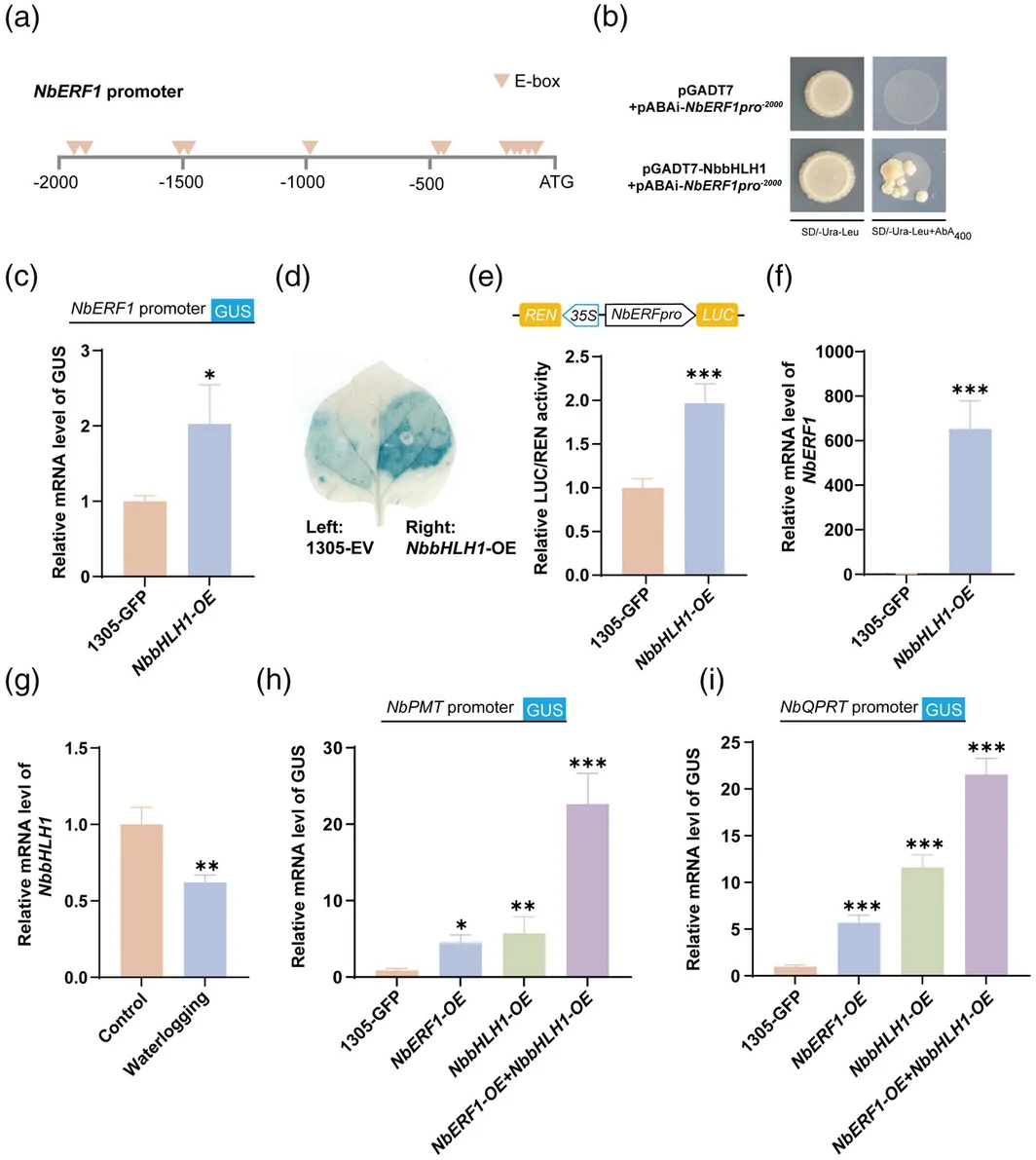
NbbHLH1 binds to the *NbERF1* promoter to indirectly regulate *NbPMT* and *NbQPRT*. (a) Sequence analysis of the *NbERF1* gene promoter. The inverted triangle indicates an E‐box. (b) Y1H assay showing the interaction of NbbHLH1 with the promoter of *NbERF1*. pAbAi‐*NbERF1pro* and pGADT7‐NbbHLH1 co‐transformed yeast grew on SD/Leu medium with or without AbA (400 ng/mL). Empty pGADT7 served as a control. (c, d) Validation of the interaction between NbbHLH1 and the *NbERF1* promoter by GUS reporter gene expression (c) and histochemical staining (d). (e) Expression levels of NbERF1 upon transient expression of a TRV‐EV (control) or TRV‐*NbbHLH1*. (f) Expression levels of *NbERF1* upon transient expression of a 1305‐EV (control) or 1305‐*NbbHLH1*‐GFP (*NbbHLH1*‐OE). (g) Expression levels of *NbbHLH1* in control and waterlogged *N. benthamiana* plants. (h, i) GUS reporter assays performed with the *NbPMT* (h) and *NbQPRT* promoters (i) in *N. benthamiana* plants expressing *NbERF1*, *NbbHLH1*, both factors, or an EV. Values represent mean ± SE, *n* = 8 biological replicates for (c); *n* = 7 biological replicates for (e); *n* = 8 biological replicates for (f); *n* = 12 biological replicates for (g); *n*= 6 biological replicates for (h), *n* = 8 biological replicates for (i). Student's *t*‐test (two‐tailed) was used for significance analysis. n.s., not significant; *P < 0.05, **P < 0.01, ***P < 0.001.

Interestingly, we noticed that the promoters of *NbQPRT* and *NbPMT* also contain multiple HLH TF binding sites (Figure S14a). Y1H confirmed NbbHLH1 binds to both promoters of *NbQPRT* and *NbPMT* (Figure S14b,c). Consistent with this, LUC and GUS assays demonstrated functional transactivation (Figure S14d–i). Also, transient expression of *NbbHLH1* significantly elevated the transcript levels of both *NbPMT* and *NbQPRT* (Figure S14j,k). We further examined the combined effects of *NbbHLH1* and *NbERF1* on *NbPMT* or *NbQPRT* promoters. Our results showed that both TFs independently enhanced promoter activity, and their coexpression resulted in significantly greater activation than either TF alone (Figure 5h,i; Figure S15). Collectively, these findings demonstrate that *NbbHLH1* regulates nicotine biosynthesis through both direct and indirect mechanisms. It directly binds to the promoters of *NbPMT* and *NbQPRT* to activate their expression and indirectly affects their expression by upregulating *NbERF1*. Together, this dual regulatory mechanism highlights the critical role of *NbbHLH1* in coordinating nicotine biosynthesis.

### Waterlogging attenuates the expression of *NbbHLH1* and *NbERF1* by decreasing the synthesis of JA

In Nicotiana species, nicotine is synthesized in the roots and transported via the vascular system to the leaves, where it accumulates and acts as a major anti‐herbivore defense compound (Kajikawa et al., 2017). Given that JA is a key positive regulator of nicotine biosynthesis during plant–herbivore interactions (Halitschke & Baldwin, 2003; Shoji et al., 2008), we investigated whether waterlogging suppresses nicotine levels through the root JA signaling pathway. While the JA and JA‐Ile levels in leaves remained unchanged under waterlogging (Figure S1a), root tissues exhibited a significant reduction in JA and JA‐Ile content, accompanied by a marked decline in the JA precursor OPDA (Figure 6a–c). The downregulation of the JA biosynthesis gene *NbAOS* and the JA signaling gene *NbCOI* (Figure 6d), coupled with reduced levels of JA metabolites, indicates that waterlogging suppresses the JA pathway in roots. Critically, exogenous JA treatment robustly induced the expression of *NbERF1* and *NbbHLH1* (Figure 6e), positioning these transcription factors as downstream effectors of JA signaling.

**Figure 6:**
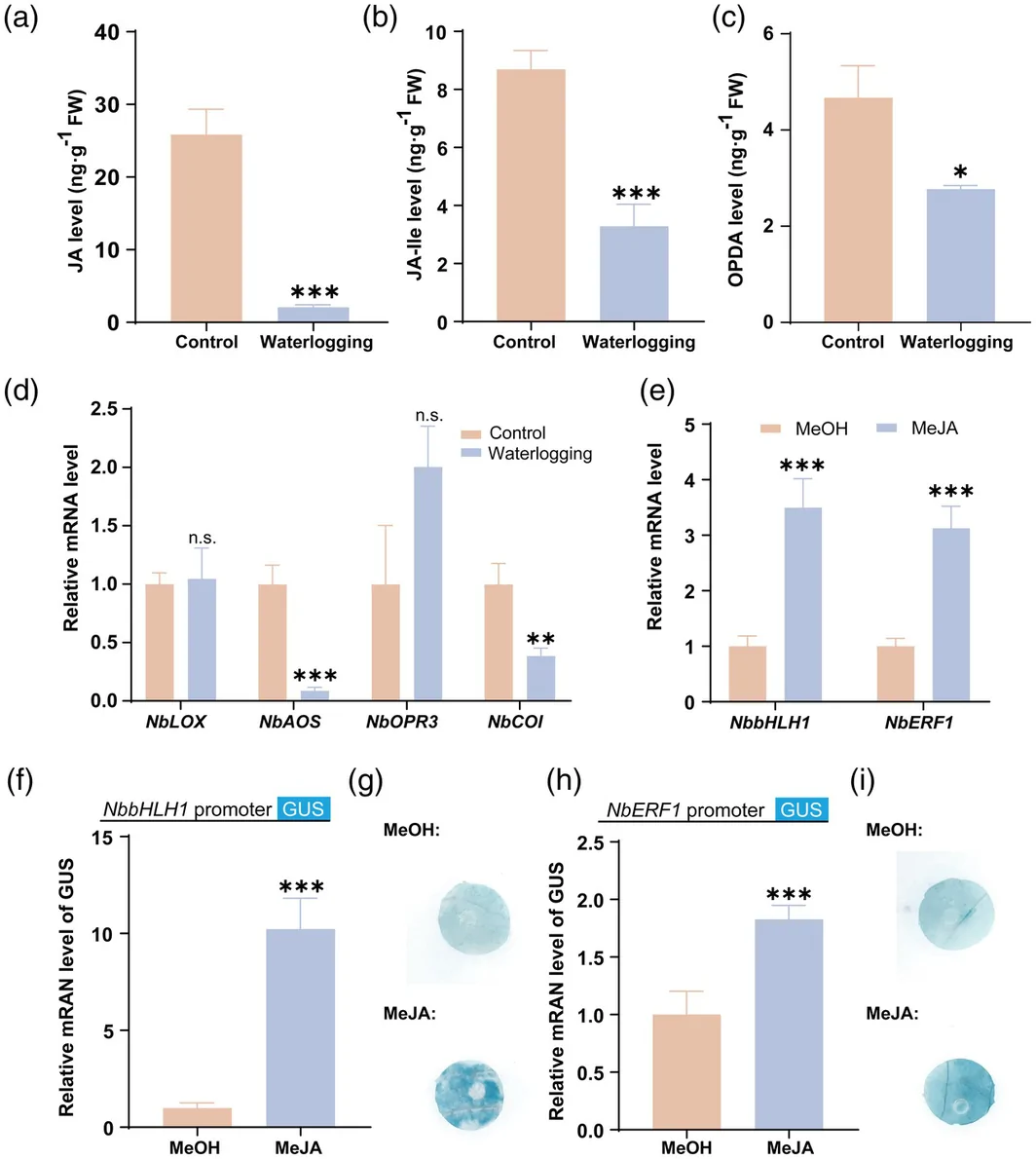
Waterlogging reduces JA levels and downregulates the expression of JA biosynthetic genes in roots. (a) JA levels in roots of control and flooded *N. benthamiana* plants. (b) JA‐Ile levels in roots of control and waterlogged *N. benthamiana* plants. (c) OPDA levels in leaves of control and waterlogged *N. benthamiana* plants. (d) Expression levels of JA biosynthetic genes in roots of well‐watered (control) and 7‐day waterlogged *N. benthamiana* plants, as determined by qRT‐PCR. (e) *NbERF1* and *NbbHLH1* expression levels in MeOH‐ or MeJA‐treated (100 μM). (f, g) GUS reporter gene expression (f) and histochemical staining (g) of the *NbbHLH1pro::GUS* construct in transiently transformed *N. benthamiana* plants after treatment with MeOH or MeJA (100 μM). (h, i) GUS reporter gene expression (h) and histochemical staining (i) of the *NbERF1pro*::*GUS* construct in transiently transformed *N. benthamiana* plants after treatment with MeOH or MeJA (100 μM). Values represent mean ± SE, *n* = 4–5 biological replicates for (a), (b), and (c) (each replicate consisted of a pool); *n* = 8 biological replicates for (d); *n* = 6–9 biological replicates for (e); *n* = 8 biological replicates for (f); *n* = 11 biological replicates for (h). Student's *t*‐test (two‐tailed) was used for significance analysis. n.s., not significant; *P < 0.05, **P < 0.01, ***P < 0.001.

To investigate whether JA directly activates the transcription of *NbERF1* and *NbbHLH1*, we conducted transient expression assays in *N. benthamiana* using *NbERF1pro*::GUS and *NbbHLH1pro*::GUS reporter constructs. Notably, MeJA treatment induced a significant increase in GUS activity compared to water‐treated controls (Figure 6f–i). Consistent with this activation pattern, dual‐luciferase assays revealed a comparable upward trend in transcriptional activity (Figure S16). These findings unequivocally demonstrate that JA signaling directly upregulates *NbERF1* and *NbbHLH1* expression. Combined with our earlier observation that waterlogging drastically reduces root JA levels, we conclude that waterlogging‐mediated JA deficiency suppresses the transcription of *NbERF1* and *NbbHLH1*. This suppression cascades to their downstream targets *NbPMT* and *NbQPRT*, ultimately diminishing nicotine biosynthesis and compromising herbivore resistance.

### JA‐ *NbbHLH1* ‐ *NbERF1* module has negative effects on plant waterlogging tolerance

To investigate why plants downregulate *NbERF1* during waterlogging and whether this downregulation contributes to waterlogging tolerance, we compared the responses of RNAi‐*NbERF1* and p35S::*NbERF1* plants under waterlogging conditions. Interestingly, we observed that while no significant differences were observed between the RNAi‐*NbERF1* and wild‐type (WT) plants under normal conditions (Figure 4a), RNAi‐*NbERF1* lines exhibited enhanced waterlogging tolerance compared to the WT plants (Figure 7a,b). After waterlogging, quantitative analysis of growth parameters revealed that both plant height and aboveground fresh weight were significantly greater in the RNAi‐*NbERF1* lines than in the WT plants (Figure 7c,d). Consistent with the shoot phenotypes, the root length and underground fresh weight of *RNAi‐NbERF1* plants were also significantly higher than those of the WT after 7 days of waterlogging treatment (Figure 7e,f). In contrast, under waterlogging conditions, p35S::*NbERF1* overexpression plants performed worse than the WT, showing reductions in plant height, aboveground fresh weight, root length, and underground fresh weight (Figure 7g–l). These results demonstrate that NbERF1 negatively regulates plant growth performance under waterlogging stress, indicating its role as a suppressor of waterlogging tolerance.

**Figure 7:**
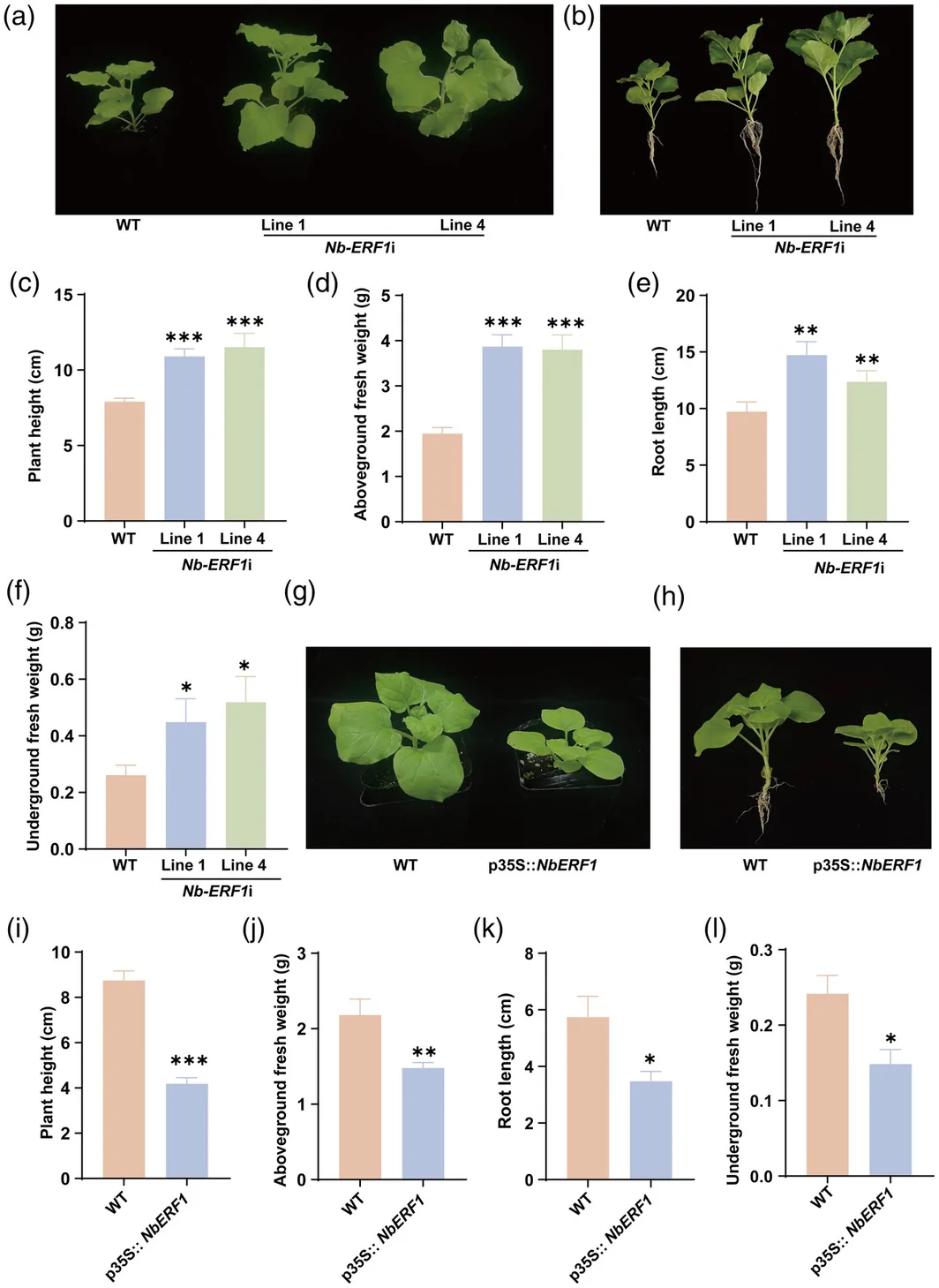
NbERF1 negatively regulates plant tolerance to waterlogging stress. (a, b) The phenotype diagram of WT and *Nb‐ERF1*i transgenic *N. benthamiana* plants after waterlogging treatment. (c) Plant height of WT and *Nb‐ERF1*i transgenic *N. benthamiana* plants after waterlogging treatment. (d) Aboveground fresh weight of WT and RNAi‐*NbERF1* transgenic *N. benthamiana* plants after waterlogging treatment. (e) Root length of WT and *Nb‐ERF1*i transgenic *N. benthamiana* plants after waterlogging treatment. (f) Underground fresh weight of WT and *Nb‐ERF1*i transgenic *N. benthamiana* plants after waterlogging treatment. (g, h) The phenotype diagram of WT and p35S*::NbERF1* transgenic *N. benthamiana* plants after waterlogging treatment. (i–l) Plant height (i), aboveground fresh weight (j), root length (k), and underground fresh weight (l) of WT and p35S*::NbERF1* transgenic *N. benthamiana* plants after waterlogging treatment. Values represent mean ± SE, *n* = 6–9 biological replicates for (c–f); *n* = 8–9 biological replicates for (i–l); Student's *t*‐test (two‐tailed) was used for significance analysis. *P < 0.05, **P < 0.01, ***P < 0.001.

Given that JA and NbbHLH1 act as upstream regulators of *NbERF1* transcription, we further examined their roles in plant growth during waterlogging stress. We found that TRV‐*NbbHLH1 N. benthamiana* plants displayed enhanced root growth under waterlogging compared to the TRV‐EV controls, although no significant differences were detected in plant height or aboveground fresh weight (Figure S17). Additionally, the JA application was found to inhibit root growth (Figure S18). Overall, these findings indicate that the JA‐NbbHLH1‐NbERF1 module negatively regulates plant growth and biomass accumulation during waterlogging stress, consistent with its function as a negative modulator of waterlogging tolerance.

## DISCUSSION

In this study, we demonstrated that waterlogging stress disrupts the synthesis of JA in plant roots, thereby suppressing the bHLH1‐ERF1 transcriptional regulatory cascade. This, in turn, affects the expression of the downstream key nicotine biosynthetic genes *NbPMT* and *NbQPRT*, ultimately reducing the accumulation of the anti‐insect alkaloid nicotine (Figure 8). These findings highlight the intricate interplay between abiotic and biotic stress responses, revealing how flood stress can compromise plant defense mechanisms and increase vulnerability to insect herbivory.

**Figure 8:**
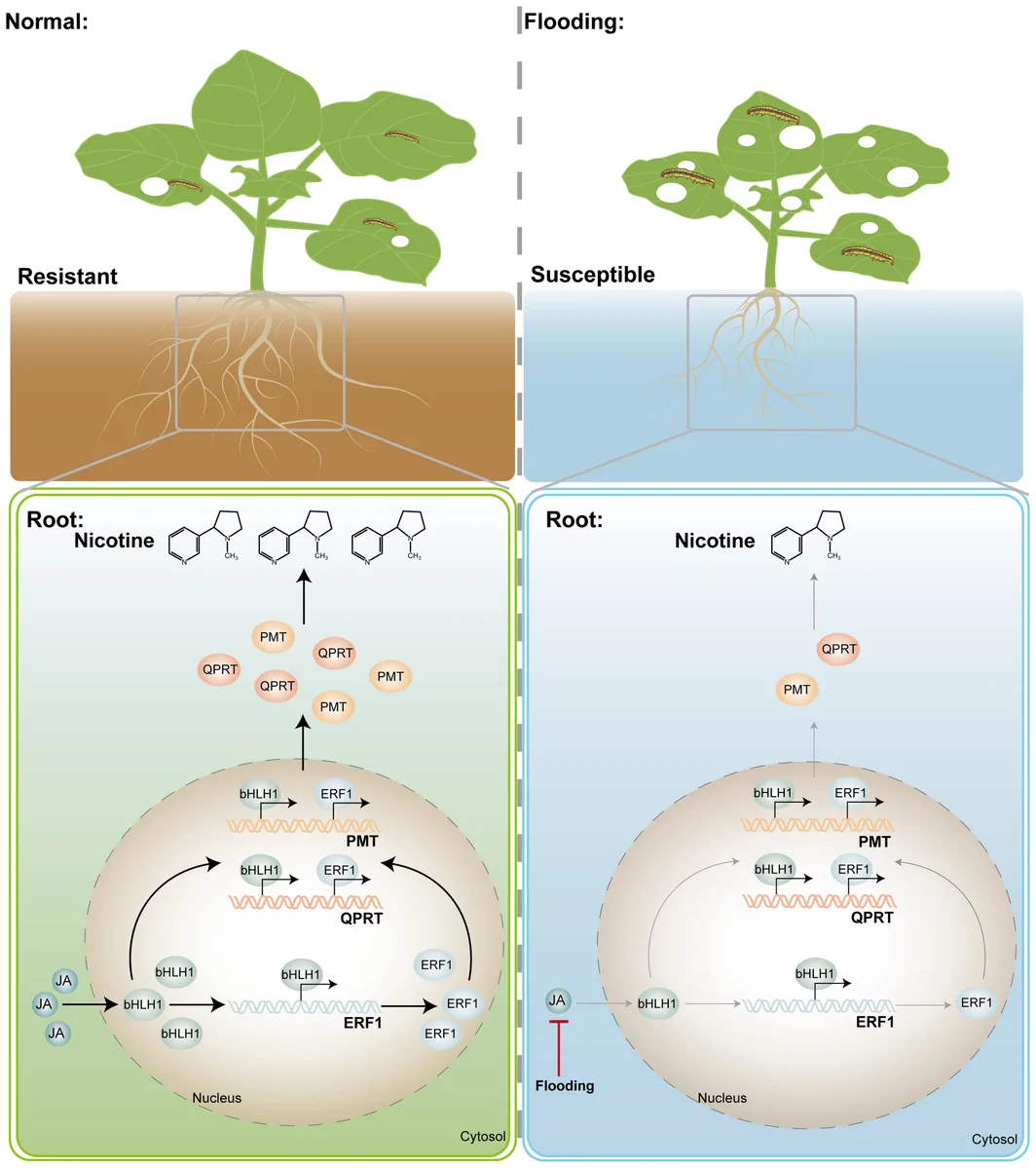
Waterlogging stress attenuates nicotine accumulation by suppressing the JA‐*NbbHLH1*‐*NbERF1* transcriptional cascade. Under normal conditions, root jasmonic acid (JA) signaling activates the expression of the transcription factor *NbbHLH1*, which in turn directly activates *NbERF1*. Both NbbHLH1 and NbERF1 bind to and transactivate the promoters of the key nicotine biosynthetic genes, *NbPMT* and *NbQPRT*, leading to high nicotine accumulation in the leaves. This confers resistance to chewing herbivores. Under waterlogging stress, root JA levels are significantly reduced. This reduction in JA signaling leads to decreased *NbbHLH1* expression, which in turn downregulates *NbERF1*. The collective downregulation of these transcription factors lowers the expression of *NbPMT* and *NbQPRT*, thereby decreasing nicotine biosynthesis and contributing to herbivore susceptibility. In the root schematic, thick black arrows indicate strong activation or signaling, while thin gray arrows represent attenuated activation or signaling under waterlogging stress. And the red blunted arrow denotes suppression.

Previous studies have reported that waterlogging stress suppresses plant anti‐insect defense and exacerbates losses in waterlogged crops (Nakamura et al., 2005; Schroeder & Lindelöw, 2012; Xie et al., 2018). However, the underlying mechanisms have not been fully elucidated, hindering the development of effective strategies for enhancing crop resistance under flood conditions. Previous studies have shown that waterlogging can enhance herbivore resistance in maize by promoting SA‐mediated flavonoid accumulation (Gorman et al., 2025). In contrast, our study found that waterlogging reduces herbivore resistance in *N. benthamiana* through suppression of nicotine biosynthesis. This discrepancy likely arises from two factors. First, plant responses to waterlogging are often species‐specific. Second, waterlogging primarily affects roots—the key site of nicotine biosynthesis in Solanaceous plants (Dawson, 1941), which compromises their anti‐herbivore defense. Consistent with this hypothesis, a previous study also found that waterlogging decreases nicotine content in *Nicotiana tabacum* L. (Chen et al., 2025). In exploring the molecular basis of this phenomenon, we observed that waterlogging stress inhibited the accumulation of nicotine by repressing the expression of key genes *NbPMT* and *NbQPRT* involved in nicotine biosynthesis in roots (Figure 2c). To further elucidate the transcriptional regulatory network involved, we screened for upstream regulators using Y1H assays. Consistent with previous studies on the role of ERFs in tobacco (Sears et al., 2014), our research reveals that the TF *NbERF1* in *N. benthamiana* binds to the promoters of key nicotine biosynthetic enzymes *NbPMT* and *NbQPRT*, activating their expression to enhance nicotine accumulation and defense against caterpillars (Figures 3, 4; Figure S5). This finding enriches the current understanding of nicotine synthesis regulation in *N. benthamiana* and highlights the crucial role of ERF TFs in plant defense against pests.

Although members of the ERF transcription factor family have been implicated in nicotine regulation in Solanaceous plants (Sears et al., 2014), the precise transcriptional mechanisms controlling their activity remain poorly understood. Our study demonstrates that *NbbHLH1* promotes the nicotine biosynthetic pathway in *N. benthamiana* by inducing the expression of *NbERF1*, which subsequently enhances nicotine accumulation and insect resistance (Figures 5 and 6). In line with previous studies (Todd et al., 2010), we found that NbbHLH1 can directly activate the expression of the nicotine biosynthetic genes *NbPMT* and *NbQPRT* (Figure S14). By integrating these findings, our work reveals a sophisticated bHLH1‐ERF1 regulatory network that employs a multifaceted strategy for nicotine control in *N. benthamiana*. Notably, NbERF1 shares high sequence homology with NtERF189 and NtERF199 in *Nicotiana tabacum* (Figure S3), which is known to be co‐expressed with the master regulator *NtMYC2* (a bHLH member) during nicotine induction (Shoji & Hashimoto, 2011). Our findings provide a plausible mechanism suggesting that this bHLH‐ERF hierarchical structure is an evolutionarily conserved module across Solanaceous species to control nicotine metabolism. Future studies should investigate whether this bHLH‐ERF hierarchical structure serves as a universal regulatory blueprint for other specialized metabolites beyond nicotine, such as phenylpropanoids or terpenoids. However, our research only illustrated the regulation of nicotine synthesis from the perspective of transcriptional activation of promoters, whereas many studies have identified transcription factor interactions at the protein level. For example, in wheat, TaERF87 physically interacts with TaAKS1, and they bind to the GCC‐box and E‐box elements in the promoters of downstream genes *TaP5CS1* and *TaP5CR1*, respectively, to synergistically activate their transcription, promote proline biosynthesis, and enhance drought tolerance (Du et al., 2023). Under cold stress, VvbHLH30 positively regulates ethylene biosynthesis by interacting with VvERF70 and binding to its promoter (Hou et al., 2026). In the future, more studies could adopt a protein–protein interaction perspective to gain deeper insights into the upstream regulatory mechanisms of nicotine biosynthesis.

Building on the complex regulatory network involving *NbbHLH1* and *NbERF1*, we next investigated the upstream signals that modulate this hierarchy, particularly under waterlogging stress. JA has been firmly established as a central positive regulator of nicotine biosynthesis (Bai et al., 2022; Sui et al., 2021). Here, we further dissected the JA‐regulated transcriptional network controlling nicotine biosynthesis and examined its modulation by waterlogging stress. While no significant change in JA content was detected in leaves (Figure S1a), root JA levels were markedly downregulated after waterlogging (Figure 6a). Given that nicotine is synthesized in the roots and its production is known to be promoted by JA signaling (De Geyter et al., 2012; Ohnmeiss et al., 1997), we propose that the waterlogging‐induced reduction in root JA underlies the decrease in leaf nicotine accumulation. These results reveal a metabolic mechanism by which waterlogging compromises plant defense, underscoring the importance of root JA in regulating nicotine‐based resistance. We further demonstrated that JA acts as a master regulator of the bHLH1‐ERF1 cascade. Exogenous JA application simultaneously induced the expression of both *NbbHLH1* and *NbERF1* (Figure 6e–i), indicating that JA controls this transcriptional hierarchy. Consequently, under waterlogging stress, JA deficiency leads to coordinated downregulation of both transcription factors, effectively dismantling the nicotine biosynthetic regulatory cascade (Figures 2a,c and 6). These findings establish JA signaling as the central orchestrator of nicotine biosynthesis via this hierarchical network and elucidate how waterlogging disrupts defense by suppressing JA accumulation.

The observed waterlogging‐induced suppression of the JA‐mediated defense pathway suggests a potential resource allocation trade‐off, where plants prioritize immediate survival to waterlogging stress over defense against herbivores. This concept aligns with growing evidence that plants prioritize resource allocation toward essential growth and survival processes over defense mechanisms when facing combined stresses (Kim et al., 2022; Lee et al., 2020; Yao et al., 2023). However, the specific signaling pathways that mediate this trade‐off remain poorly understood. In our study, we found that waterlogging stress led to a coordinated downregulation of JA levels and the expression of transcription factors *NbERF1* and *NbbHLH1* (Figures 3m, 5i and 6a). Meanwhile, *Nb‐ERF1*i plants and *NbbHLH1*‐silenced plants performed better under waterlogging conditions (Figure 7a–f; Figure S10), whereas p35S::*NbERF1* overexpression lines performed worse (Figure 7g–l). These findings elucidate the genetic mechanism underlying a critical stress trade‐off, providing a foundation for breeding strategies aimed at uncoupling herbivore resistance from waterlogging sensitivity, such as by employing inducible promoters to activate anti‐herbivore responses only when needed. In addition, a key remaining question is whether the negative impact of JA and *NbbHLH1* on waterlogging tolerance operates entirely depending on *NbERF1*. A deeper understanding of this regulatory mechanism will not only elucidate how plants respond to waterlogging but also provide fundamental insights into trade‐off principles in resource allocation.

Field crops are often subjected to multiple environmental stresses simultaneously. Therefore, understanding how plants respond to combined stresses is of great significance for ensuring crop yield and quality. This study reveals how waterlogging stress increases plant vulnerability to herbivorous insects by disarming their chemical defense through inhibiting the JA‐mediated bHLH1‐ERF1 transcriptional axis. These findings not only elucidate the resource trade‐off mechanism between flood survival and herbivore resistance in plants, but also provide new targets for breeding crop varieties with enhanced resilience to both waterlogging and insect herbivory through genetic improvement.

## MATERIAL AND METHODS

### Plant cultivation and waterlogging treatment

Seeds of *Nicotiana benthamiana* plants (accessions LAB and H2B‐RFP) were provided by Dr. Xueping Zhou from the Institute of Biotechnology, Zhejiang University. Overexpression and RNAi lines of *N. benthamiana* (p35S::*NbERF1* and RNAi‐*NbERF1*) were generated by Biorun Co., Ltd. (China). The *N. benthamiana* seeds were sown in a sterile substrate composed of peat moss, perlite, and vermiculite (5:3:1, v/v/v) within seed starter trays. The trays were placed in climate‐controlled chambers under a 14/10 h light/dark photoperiod, with temperature maintained at 26 ± 1°C and relative humidity regulated to 65 ± 10%. At 14‐day post‐sowing, seedlings exhibiting fully expanded cotyledons and 2–3 true leaves were transplanted into individual plastic pots containing the same substrate.

For waterlogging treatments, 14‐day post‐transplantation plants (28‐day‐old seedlings) were transferred to plastic basins. Water was gradually added until the soil surface and root zone were submerged, while ensuring the basal stem remained unimmersed. To stabilize pots during waterlogging, stones (0.3–0.5 cm in diameter) were evenly distributed across the soil surface. Waterlogging stress was applied continuously for 7 days, with control plants maintained under standard irrigation. All experiments were conducted under consistent environmental conditions to minimize variability.

### Insect bioassay

For the insect bioassay, *Helicoverpa armigera*, *Spodoptera litura*, and *Spodoptera frugiperda* second‐instar larvae were purchased from Jiyuan Baiyun Industry Co., Ltd. (Henan, China). Before feeding assays, uniform second‐instar larvae were selected and starved for 24 h. Larvae were placed on both control and waterlogging‐treated plants. Two larvae were placed on each plant, and their weights were recorded after 48 h. The assay was performed following a previously described method, with the feeding duration shortened to 48 h to prevent herbivore starvation caused by rapid consumption of waterlogging‐treated plants (Wu et al., 2024).

### MeJA treatment

Root treatment for analysis gene expression: The experimental method was based on a previous study (Nakagawa and Kawaguchi, 2006). For the treatment group, *N. benthamiana* plants at 14 days after transplanting received 5 mL of 100 μM methyl jasmonate (MeJA) (CAS:39924‐52‐2; Merck KGaA Co., Ltd, Shanghai, China) dissolved in distilled deionized water containing 0.1% (v/v) methanol. The solution was applied directly to the root zone. Control plants were administered an equal volume of 0.1% methanol in distilled deionized water without MeJA. Samples were collected after 48 h.

Root treatment for measuring root length: The experimental method was based on a previous study, with modifications to the concentration based on plant growth status (Duric et al., 2023). *N. benthamiana* seeds were initially grown on full‐strength Murashige and Skoog (MS) medium for 7 days, and then transferred to MS medium supplemented with MeJA at a final concentration of 10 μM and maintained for an additional 3 days. Control plants were transferred to MS medium containing an equal volume of 0.1% methanol in distilled deionized water.

Leaf treatment for GUS and dual‐luciferase assays: The method and concentration of MeJA application were described previously (Wu et al., 2019). *N. benthamiana* plants 14 days after transplanting were evenly sprayed with 100 μM MeJA (Merck, Shanghai, China). The MeJA stock solution was prepared in methanol, and the working solution contained a final concentration of 0.1% (v/v) methanol. Control plants were sprayed with an equal volume of distilled deionized water containing 0.1% MeJA‐free methanol. Leaf samples were collected after 48 h.

### Measurement of SA and JA

The measurement of SA and JA content was conducted following the method outlined in a previous study (Zhao et al., 2025). *N. benthamiana* plants were sampled following a 7‐day waterlogging treatment. Approximately 150 mg of leaf tissue per replicate was flash‐frozen, pulverized in liquid nitrogen, and stored at −80°C. For extraction, 1 mL of ethyl acetate containing internal standards (200 ng of D6‐JA, 13C2‐JA‐Ile, and D4‐SA) was added, followed by 10‐minute vortexing and centrifugation (12 000 rpm, 10 min, 4°C). The supernatant was collected, and the pellet was re‐extracted with 0.5 mL ethyl acetate. The combined supernatants were dried in a vacuum concentrator (Eppendorf). The residue was reconstituted in 0.5 mL of 70% methanol, centrifuged, and the resulting supernatant was analyzed by HPLC–MS/MS (Shimadzu LCMS‐8040). Chromatographic separation was performed using a Shim‐pack XR‐ODS III column (75 × 2 mm, 1.6 μm) with a gradient of 0.05% formic acid/5 mM ammonium formate (A) and methanol (B) at 0.3 mL/min. Detection was performed in negative ionization mode with a 10 μL injection volume.

### Measurement of nicotine

For nicotine content measurement under waterlogging conditions, *N. benthamiana* plants were sampled after 7 days of waterlogging treatment. For nicotine content analysis in wild‐type (WT) and transgenic lines, plants were sampled at 14 days after transplanting.

A leaf half without a midvein (100 mg) was harvested and frozen in liquid N_2_. Leaf material was ground frozen in a 2 mL microcentrifuge tube. The samples were extracted by shaking the tubes vigorously for 2 h with 1.5 mL of 40% aqueous MeOH containing 0.5% acetic acid. After centrifugation (10 min, 13 000 rpm), the supernatant was transferred into a sample vial and diluted 10 times for HPLC–MS/MS analysis. The chromatographic separations of all the samples were performed using an UHPLC system (SCIEX, USA). An ACQUITY UPLC BEH C18 column (1.8 μm × 2.1 mm × 50 mm) (Waters, USA) was used for the reversed‐phase separation. The column oven was maintained at 40°C and the flow rate was 200 μL/min. The mobile phase consisted of solvent A (0.1% formic acid) (HPLC grade, Xiya Reagent, China) and solvent B (acetonitrile) (HPLC grade, Merck, USA). The gradient elution procedures were set as follows: 0 min 5% B; 1 min 5%; 4 min 95%; 5 min 95% B; 5.2 min 5%; post‐time is 2 min. The injection volume for each sample was 2 μL and the automatic sampler temperature is 4°C. LC‐MS/MS analyses were performed using an SCIEX Triple Quad 4500 (SCIEX, USA) equipped with an electrospray ionization (ESI) source, operated in the positive ion multiple reaction monitoring (MRM) mode. SCIEX Workstation was used for data acquisition and processing.

### Determination of lignin content

*N. benthamiana* plants subjected to 7 days of waterlogging treatment, along with control plants, were used for analysis. Lignin content was determined using the acetyl bromide (AcBr) method as previously described (Ji et al., 2024). The third and fifth leaves from the base of each plant were harvested and dried at 37°C for 3 days. The dried leaf material was ground and passed through a 50‐mesh sieve. About 5 mg of the resulting powder was accurately weighed into a 1.5 mL microcentrifuge tube, and a solution of acetyl bromide in acetic acid was added. The mixture was incubated at 80°C for 40 min. After centrifugation, the supernatant was collected, and total lignin content was determined.

### Determination of trichome density

Trichome density was determined as described previously (Wang et al., 2025).

*N. benthamiana* plants subjected to 7 days of waterlogging treatment and control plants were used for trichome density analysis. Trichome density was determined by counting trichomes on both the adaxial and abaxial leaf surfaces using a Zeiss Stemi 2000‐C stereomicroscope. From each plant, the third and fifth leaves from the base (with the basal leaf designated as leaf 1) were collected. Three 25 mm^2^ areas per leaf were examined, and ten biological replicates were analyzed per treatment. Trichomes were counted manually, and trichome density was calculated as the number of trichomes per mm^2^ of leaf area.

### RNA extraction and RT‐qPCR analysis

Total RNA was isolated from plant tissues using AG RNAex Pro reagent (Accurate Biology, Changsha, China). For mRNA analysis, single‐stranded complementary DNA (cDNA) was synthesized from 2 μg of DNase I‐treated total RNA using the Evo M‐MLV RT Kit with gDNA Clean for qPCR Ver.2 (Accurate Biology, Changsha, China). Quantitative PCR amplification was carried out on a Bio‐Rad CFX96 Real‐Time PCR Detection System (Bio‐Rad Laboratories, Hercules, CA, USA) with SYBR Green‐based chemistry. Reactions contained 10 μL of 2× SYBR Green Pro Taq HS Premix (Accurate Biology, Changsha, China), 0.4 μM of gene‐specific forward and reverse primers (Table S1), 2 μL of diluted cDNA template, and nuclease‐free water to a final volume of 20 μL. Expression levels were quantified using the comparative threshold cycle (2^−ΔCT^) method. The *N. benthamiana* housekeeping gene *NbACTIN* served as the internal control for gene expression analysis. The primers of RT‐qPCR for nicotine synthesis genes and transcription factors were referred to previous studies (Todd et al., 2010). Primers used in this study are listed in Table S1.

### Virus‐induced gene silencing assay

The virus‐induced gene silencing (VIGS) assay was carried out as previously described (Liu & Page, 2008). Approximately 500‐bp fragments of the target genes from *N. benthamiana* were designed by the VIGS tool on the Sol Genomics Network (https://vigs.solgenomics.net/). The fragment sequences of *NbERF1* and *NbbHLH1* were amplified from *N. benthamiana* root cDNA using the I‐5™ 2× High‐Fidelity Master Mix (MCLAB, San Francisco, CA, USA) and cloned into the TRV2 vector via homologous recombination using the NovoRec Plus Recombinase Kit (Novoprotein, Shanghai, China). The recombinant plasmids were then transformed into *Agrobacterium tumefaciens* strain EHA105 by chemical transformation. Following cultivation, *A*. *tumefaciens* cells were harvested, resuspended, and adjusted to an OD600 of 0.2. Cultures containing pTRV1 were mixed 1:1 (v/v) with those carrying pTRV2‐derived constructs: pTRV2‐EV (empty vector control), pTRV2‐*NbPMT*, pTRV2‐*NbQPRT*, pTRV2‐*NbbHLH1*, or pTRV2‐*NbERF1*. The mixed cultures were pressure‐infiltrated into the abaxial surface of *N. benthamiana* plants at 14 days after transplanting. Infiltrated plants were maintained under controlled climate chamber conditions. Fourteen days later, the treated plants were used for follow‐up experiments, including bioassays and sample collection. Primers used in this study are listed in Table S1.

### Yeast one‐hybrid assays

To identify proteins that interact with the promoters of *NbPMT*, *NbQPRT*, and *NbERF1*, the Yeast User Manual PT4087‐1 (Clontech, Shiga, Japan) was employed. Approximately 2‐kb promoter fragments of NbPMT, NbQPRT, and NbERF1 were amplified by PCR from *N. benthamiana* genomic DNA using gene‐specific primers (Table S1) and KOD FX Neo polymerase (Toyobo, Osaka, Japan). The purified PCR products were cloned into the pAbAi vector via homologous recombination. The full‐length coding sequences of *NbERF1* and *NbbHLH1* were amplified from *N. benthamiana* root cDNA using the I‐5™ 2× High‐Fidelity Master Mix and cloned into the pGADT7 AD using homologous recombination. These recombinant vectors were transformed into Y1H Gold Yeast Competent Cells (Shanghai ViDi Biotech Co., Shanghai, China). Yeast cells were cultured for 3–5 days at 28°C on SD/‐Ura/−Leu medium, with or without the addition of Aureobasidin A (AbA) to prevent self‐activation.

### Histochemical GUS activity assays

To investigate transcriptional regulation, each transcription factor (TF) was employed as an effector in a GUS reporter system. The 2000‐bp promoter regions of *NbERF1*, *NbPMT*, and *NbQPRT* were amplified from *N. benthamiana genomic* DNA using gene‐specific primers (Table S1) and KOD FX Neo polymerase and cloned into the pBI121 vector upstream of the β‐glucuronidase (GUS) reporter gene via homologous recombination. For effector construction, the full‐length coding sequences of NbbHLH1 and NbERF1 were amplified from *N. benthamiana* root cDNA using the I‐5™ 2 × High‐Fidelity Master Mix and inserted into the pCAMBIA1305 vector to generate C‐terminal in‐frame fusions with GFP, yielding pCAMBIA1305‐*NbbHLH1*‐GFP and pCAMBIA1305‐*NbERF1*‐GFP. The empty pCAMBIA1305‐GFP vector served as a negative control. All constructs were introduced into *A. tumefaciens* strain EHA105 for transient expression assays. Reporter and effector constructs were co‐infiltrated into *N. benthamiana* leaves. GUS activity was quantified fluorometrically 48‐hour post‐infiltration to assess the transcriptional activation effect. Primers used in this study are listed in Table S1.

### Dual‐luciferase assays

The 2000‐bp promoter regions of *NbERF1*, *NbPMT*, and *NbQPRT* were amplified from *N. benthamiana* genomic DNA using gene‐specific primers (Table S1) and KOD FX Neo polymerase and cloned into the reporter vector pGreenII 0800‐LUC upstream of the firefly luciferase (LUC) gene via homologous recombination. The following effector constructs were used: pCAMBIA1305‐*NbERF1*‐GFP, pCAMBIA1305‐*NbbHLH1*‐GFP, and the empty vector pCAMBIA1305 (as a negative control). All constructs were introduced into *A. tumefaciens* strain EHA105 (pSoup) for transient expression. In this dual‐luciferase system, LUC expression was driven by the target promoters, while Renilla luciferase (REN), driven by the constitutive CaMV 35S promoter, served as an internal reference. Reporter and effector constructs were co‐infiltrated into *N. benthamiana* leaves via *Agrobacterium*‐mediated transformation. After 48 hours of incubation, LUC and REN activities were quantified using the Dual‐Luciferase Reporter Assay Kit (Vazyme, Nanjing, China) on a FlexStation 3 Multi‐Mode Microplate Reader (Molecular Devices, San Jose, CA, USA). Relative transcriptional activation was calculated by normalizing LUC signals to REN values (LUC: REN ratio).

### Subcellular localization

For subcellular localization studies, *A.tumefaciens* strain EHA105 harboring the following constructs was used: pCAMBIA1305‐*NbbHLH1*‐GFP, pCAMBIA1305‐*NbERF1*‐GFP, and the empty vector pCAMBIA1305‐GFP. These constructs were agroinfiltrated into leaves of *N. benthamiana* H2B‐RFP transgenic plants, which constitutively express a nuclear‐localized red fluorescent protein (RFP) fused to histone H2B. GFP fluorescence signals were observed 48 hours post‐infiltration using a confocal laser scanning microscope (Zeiss LSM710, Oberkochen, Germany).

### Data analysis

Data analysis was performed using IBM SPSS Statistics 22.0, and results were visualized with GraphPad Prism 9. For pairwise comparisons, statistical significance was assessed using a two‐tailed Student's *t*‐test.

### Accession numbers

NbbHLH1 (GQ859152), NbERF1 (GQ859157), NbPMT (Niben101Scf06783g00003.1), NbQPRT (Niben101Scf03687g01001.1).

## Author Contributions

YDX, WHH, JXW, and JJW conceived and designed the experiments. YDX, JXW, YJC, and CZ performed the experiments. YDX, JXW, and WHH analyzed the data. YDX, WHH, YQL, SSL, and XWW wrote the paper. All authors read and approved the manuscript.

## Conflict of Interest

The authors declare no conflicts of interest.

## Supporting information


**Figure S1.** Effect of waterlogging on JA levels, SA levels, trichome density, and lignin content in *N. benthamiana* plants.
**Figure S2.** Expression levels of *NbPMT* and *NbQPRT* in control and VIGS *N. benthamiana* plants.
**Figure S3.** Sequence alignment of NbERF1 with NtERF189 and NtERF199. The black line indicates the conserved AP2/ERF DNA‐binding domain.
**Figure S4.** NbERF1‐GFP nuclear localization in *N. benthamiana* H2B RFP line.
**Figure S5.** Validation of NbERF1 binding to the *NbPMT* and *NbQPRT* promoters via dual‐luciferase assays.
**Figure S6.** Y1H and GUS assays with NbERF1 and its DNA‐binding domain deletion mutant.
**Figure S7.** Y1H and GUS assays with promoter ERF‐binding site mutants in *NbPMT* and *NbQPRT*.
**Figure S8.** Expression levels of *NbERF1* in the leaves (a) and stems (b) of control and waterlogged *N. benthamiana* plants.
**Figure S9.** Confirmation of *Nb‐ERF1*i and p35S::*NbERF1* transgenic *N. benthamiana* plants.
**Figure S10.** Weight of *H. armigera* feeding on WT and transgenic *N. benthamiana* plants after waterlogging treatment.
**Figure S11.** NbbHLH1‐GFP nuclear localization in *N. benthamiana* H2B RFP line.
**Figure S12.** Validation of the interaction between NbbHLH1 and the *NbERF1* promoter by dual‐luciferase assays.
**Figure S13.** Effect of silencing *NbbHLH1* on *H. armigera* resistance in plants.
**Figure S14.** NbbHLH1 binds to the promoters of *NbPMT* and *NbQPRT* to activate their transcription and regulate plant resistance to herbivores.
**Figure S15.** Dual‐luciferase assays show the activity of the *NbPMT* promoter in plants expressing *NbERF1*, *NbbHLH1*, both factors, or an EV.
**Figure S16.** MeJA treatment upregulates the transcriptional activity of *NbbHLH1* and *NbERF1*.
**Figure S17.** *NbbHLH1* negatively regulates plant tolerance to waterlogging.
**Figure S18.** JA negatively regulates plant growth.
**Table S1.** List of primers used in this study.

## Data Availability

The data that support the findings of this study are available in the Supporting Information of this article.
