# Supplementary material for: Waterlogging stress disarms nicotine‐mediated defense to herbivores through the bHLH1‐ERF1 transcriptional switch
Source: Plant J. 2026 Nov 9;128(3):e71192. doi: 10.1111/tpj.71192 (PMC13748688; doi:10.1111/tpj.71192)
Supplement: Supplementary file 1 [file TPJ-128-0-s001.docx]

## **Supplementary Material**

**Article title:** Waterlogging stress disarms nicotine-mediated defense to herbivores through the bHLH1-ERF1 transcriptional switch

Authors: Yu-Die Xiong†, Wen-Hao Han†, Jun-Xia Wang, Jia-Jing Wang, Chan Zhao, Yu-Jie Chi, Shu-Sheng Liu, Xiao-Wei Wang*

The following Supporting Information is available for this article:

**Figure S1** Effect of waterlogging on JA levels, SA levels, trichome density, and lignin content in *N. benthamiana* plants.

**Figure S2** Expression levels of *NbPMT* and *NbQPRT* in control and VIGS *N. benthamiana* plants.

**Figure S3** Sequence alignment of NbERF1 with NtERF189 and NtERF199. The black line indicates the conserved AP2/ERF DNA-binding domain.

**Figure S4** NbERF1-GFP nuclear localization in *N. benthamiana* H2B RFP line.

**Figure S5** Validation of NbERF1 binding to the *NbPMT* and *NbQPRT* promoters via dual‑luciferase assays.

**Figure S6** Y1H and GUS assays with NbERF1 and its DNA‑binding domain deletion mutant.

**Figure S7** Y1H and GUS assays with promoter ERF‑binding site mutants in *NbPMT* and *NbQPRT*.

**Figure S8** Expression levels of *NbERF1* in the leaves (a) and stems (b) of control and waterlogged *N. benthamiana* plants.

**Figure S9** Confirmation of *Nb-ERF1*i and p35S::*NbERF1* transgenic *N. benthamiana* plants.

**Figure S10** Weight of *H. armigera* feeding on WT and transgenic *N. benthamiana* plants after waterlogging treatment.

**Figure S11** NbbHLH1-GFP nuclear localization in *N. benthamiana* H2B RFP line.

**Figure S12** Validation of the interaction between NbbHLH1 and the *NbERF1* promoter by dual-luciferase assays.

**Figure S13** Effect of silencing *NbbHLH1* on *H. armigera* resistance in plants.

**Figure S14** NbbHLH1 binds to the promoters of *NbPMT* and *NbQPRT* to activate their transcription and regulate plant resistance to herbivores.

**Figure S15** Dual-luciferase assays show the activity of the *NbPMT* promoter in plants expressing *NbERF1*, *NbbHLH1*, both factors, or an EV.

**Figure S16** MeJA treatment upregulates the transcriptional activity of *NbbHLH1* and *NbERF1*.

**Figure S17** *NbbHLH1* negatively regulates plant tolerance to waterlogging.

**Figure S18** JA negatively regulates plant growth.

**Table S1** List of primers used in this study

**Figure S1** Effect of 7-day waterlogging treatment on JA and SA levels, trichome density, and lignin content in *N. benthamiana* leaves.

(a) JA levels in leaves of control and waterlogging treatment *N. benthamiana* plants. (b) SA levels in leaves of control and waterlogged *N. benthamiana* plants. (c) Trichome density (number of trichomes per cm²) on the 3rd and 5th leaves of control and waterlogged *N. benthamiana* plants. (d) Lignin content (mg/g dry weight) in the 3rd and 5th leaves of control and waterlogged *N. benthamiana* plants. Values represent mean ± SE, n=3 biological replicates for (a and b) (each replicate is a pool of two plants); n=6 biological replicates for (c); n=4 biological replicates for (d). Student’s *t*-test (two-tailed) was used for significance analysis. n.s., not significant.

**Figure S2** Expression levels of *NbPMT* and *NbQPRT* in control and VIGS *N. benthamiana* plants.

(a) Expression levels of *NbPMT* in TRV-EV and TRV-*NbPMT* *N. benthamiana* plants. (b) Expression levels of *NbQPRT* in TRV-EV and TRV-*NbQPRT N. benthamiana* plants. Values represent mean ± SE, n=6-8 biological replicates for (a), n=6 biological replicates for (b). Student’s *t*-test (two-tailed) was used for significance analysis. n.s., not significant; **P* < 0.05, ****P* < 0.001.

**Figure S3** NbERF1-GFP nuclear localization in *N. benthamiana* H2B RFP line. 1305-EV as control. Scale bar=10 μm.

**Figure S4** Sequence alignment of NbERF1 with NtERF189 and NtERF199. The black line indicates the conserved AP2/ERF DNA-binding domain.

**Figure S5** Validation of NbERF1 binding to the *NbPMT* and *NbQPRT* promoters via dual‑luciferase assays.

Validation of the interaction between NbERF1 and the *NbPMT* (a) or *NbQPRT* (b) promoter by dual-luciferase assays. The LUC signals were normalized to REN activity. Values are mean ± SE, n=7 biological replicates for (a) and (b). Student’s *t*-test (two-tailed) was used for significance analysis. ****P* < 0.001.

**Figure S6** Y1H and GUS assays with NbERF1 and its DNA‑binding domain deletion mutant.

(a and b) Y1H assays to test the binding of NbERF1 and ΔNbERF1 to the *NbPMT* and *NbQPRT* promoters. Yeast cells co‑transformed with pGADT7‑NbERF1 or pGADT7‑ΔNbERF1, together with pAbAi‑*NbPMTpro* (a) or pAbAi‑*NbQPRTpro* (b), were cultured on SD/Leu medium supplemented with or without 350 ng/mL AbA. Empty pGADT7 served as a control. (c and d) GUS reporter assays to test the transactivation of the *NbPMT* (c) and *NbQPRT* (d) promoters by NbERF1 and ΔNbERF1.Values are mean ± SE, n=9-10 for biological replicates for (c), n=5-8 biological replicates for (d). Student’s *t*-test (two-tailed) was used for significance analysis. n.s., not significant, **P* < 0.05, ***P* < 0.01.

**Figure S7** Y1H and GUS assays with promoter ERF‑binding site mutants in *NbPMT* and *NbQPRT*.

(a) Schematic diagram of *NbPMT* promoter mutant sites; red indicates the mutated positions. (b) Y1H assays to test the binding of NbERF1 to the *NbPMT* and Δ*NbPMT* promoters. Yeast cells co-transformed with pGADT7-NbERF1 and pAbAi-*NbPMTpro* or pAbAi-Δ*NbPMTpro* were cultured on SD/Leu medium in the presence or absence of 350 ng/mL AbA. Empty pGADT7 served as a control. (c) GUS reporter assays to test the transactivation of the *NbPMT* and Δ*NbPMT* promoters by NbERF1. (d) Schematic diagram of *NbQPRT* promoter mutant sites; red indicates the mutated positions. (e) Y1H assays to test the binding of NbERF1 to the *NbQPRT* and Δ*NbQPRT* promoters. Yeast cells co-transformed with pGADT7-NbERF1 and pAbAi-*NbQPRTpro* or pAbAi-Δ*NbQPRTpro* were cultured on SD/Leu medium in the presence or absence of 350 ng/mL AbA. Empty pGADT7 served as a control. (f) GUS reporter assays to test the transactivation of the *NbQPRT* and Δ*NbQPRT* promoters by NbERF1. Student’s *t*-test (two-tailed) was used for significance analysis. Values are mean ± SE, n=6 for biological replicates for (c) and (f). n.s., not significant, **P* < 0.05, ****P* < 0.001.

**Figure S8** Expression levels of *NbERF1* in the leaves (a) and stems (b) of control and waterlogged *N. benthamiana* plants. Values are mean ± SE, n=9-10 biological replicates for (a) and (b). Student’s *t*-test (two-tailed) was used for significance analysis. n.s., not significant.

**Figure S9** Confirmation of *Nb-ERF1*i and p35S::*NbERF1* transgenic *N. benthamiana* plants.

(a) Expression levels of *NbERF1* in wild-type (WT) and *Nb-ERF1*i transgenic *N. benthamiana* plants. (b) Expression levels of *NbERF1* in WT and p35S::*NbERF1* transgenic *N. benthamiana* plants. (c) Detection of GFP-tagged NbERF1 protein expression in WT and p35S::*NbERF1* transgenic *N. benthamiana* plants by Western blot. n=6-8 biological replicates for (a and b). Student’s *t*-test (two-tailed) was used for significance analysis. ****P* < 0.001.

**Figure S10** Weight of *H. armigera* feeding on WT and transgenic *N. benthamiana* plants after waterlogging treatment.

(a) Weight of *H. armigera* feeding on WT and *Nb-ERF1*i transgenic *N. benthamiana* plants after waterlogging treatment. (b) Weight of *H. armigera* feeding on WT and p35S::*NbERF1* transgenic *N. benthamiana* plants after waterlogging treatment. Values represent mean ± SE, n=10 biological replicates for (a and b). Student’s *t*-test (two-tailed) was used for significance analysis. **P* < 0.05, ***P* < 0.01, ****P* < 0.001.

**Figure S11** NbbHLH1-GFP nuclear localization in *N. benthamiana* H2B RFP line. 1305-EV as control. Scale bar=10 μm.

**Figure S12** Validation of the interaction between NbbHLH1 and the *NbERF1* promoter by dual-luciferase assays. The LUC signals were normalized to REN activity. Values represent mean ± SE, n=10 biological replicates. Student’s *t*-test (two-tailed) was used for significance analysis. n.s., not significant; ****P* < 0.001.

**Figure S13** Effect of silencing *NbbHLH1* on *H. armigera* resistance in plants.

(a) Expression levels of *NbbHLH1* in TRV-EV and TRV-*NbbHLH1* *N. benthamiana* plants. (b) Weight of *H. armigera* after feeding on TRV-EV and TRV-*NbbHLH1 N. benthamiana*. (c) Representative images of *H. armigera* after feeding on TRV-EV and TRV-*NbbHLH1*. Values represent mean ± SE, n=8 biological replicates for (a); n=8-10 biological replicates for (b). Student’s *t*-test (two-tailed) was used for significance analysis. **P* < 0.05, ****P* < 0.001.

**Figure S14** NbbHLH1 binds to the promoters of *NbPMT* and *NbQPRT* to activate their transcription and regulate plant resistance to herbivores.

(a) Sequence analysis of *NbPMT* and *NbQPRT* promoters, with triangles marking E-box motifs. (b and c) Y1H assay showing the interaction of NbbHLH1 with the promoters of *NbPMT* and *NbQPRT*. Yeast co-transformed with pGADT7-NbbHLH1 and pAbAi-*NbPMTpro* (b) or pAbAi*-NbQPRTpro* (c) grew on SD/Leu medium with or without AbA (350 ng/mL). Empty pGADT7 served as a control. (d and e) Validation of NbbHLH1 and *NbPMT* promoter interaction by GUS reporter gene expression (d) and histochemical staining (e). (f and g) Validation of NbbHLH1 and *NbQPRT* promoter interaction by GUS reporter gene expression (f) and histochemical staining (g). (h and i) Validation of NbbHLH1 and the *NbPMT* (h) and *NbQPRT* promoter (i) interaction by dual-luciferase assays. The LUC signals were normalized to REN activity. (j and k) Expression levels of *NbPMT* (j) and *NbQPRT* (k) in plants transiently expressing a control vector or *NbbHLH1* overexpression construct. Values represent mean ± SE, n=7 biological replicates for (d and f); n=8 biological replicates for (h and i); n=4 biological replicates for (j) and (k). Student’s *t*-test (two-tailed) was used for significance analysis. **P* < 0.05, ***P* < 0.01, ****P* < 0.001.

**Figure S15** Dual-luciferase assays show the activity of the *NbPMT* promoter in plants expressing *NbERF1*, *NbbHLH1*, both factors, or an EV. The diagram shows the reporter construct. Values represent mean ± SE, n=6 biological replicates. Student’s t-test (two-tailed) was used for significance analysis. ****P* < 0.001.

**Figure S16** MeJA treatment upregulates the transcriptional activity of *NbbHLH1* and *NbERF1*.

(a and b) LUC activity of reporter constructs driven by the *NbbHLH1* (a) or *NbERF1* (b) promoter after treatment with MeOH or MeJA (100 μM). Values represent mean ± SE, n=5 biological replicates for (a and b). Student’s t-test (two-tailed) was used for significance analysis. n.s., not significant; ****P* < 0.001.

**Figure S17** *NbbHLH1* negatively regulates plant tolerance to waterlogging.

(a) Expression levels of *NbbHLH1* in TRV-EV (control) and TRV-*NbbHLH1* *N. benthamiana* plants. (b and c) Phenotypes of TRV-EV and TRV-*NbbHLH1* *N. benthamiana* plants after waterlogging treatment. (d, e, f, and g) Biometric parameters of TRV-EV and TRV-*NbbHLH1* *N. benthamiana* plants after waterlogging treatment: plant height (d), aboveground fresh weight (e), root length (f), underground fresh weight (g). Values represent mean ± SE, n=9 biological replicates for (a), (d), (e), (f) and (g). Student’s t-test (two-tailed) was used for significance analysis. n.s., not significant, **P* < 0.05, ****P* < 0.001.

**Figure S18** JA negatively regulates plant growth.

(a) Phenotypes of *N. benthamiana* treated with MeOH or 10 μM MeJA for 3 days. Scale bar=1 cm. (b) Root length of *N. benthamiana* treated with MeOH or 10 μM MeJA for 3 days. Values represent mean ± SE, n=10 biological replicates for (b). Student’s t-test (two-tailed) was used for significance analysis. n.s., not significant, ****P* < 0.001.

**Table S1. List of primers used in this study.**

| Primers | Sequences (5’-3’) |
| --- | --- |
| TRV2-NbERF1-F | CTGTGAGTAAGGTTACCGAATTCATGAATTCAGCAGATGTAACC |
| TRV2-NbERF1-R | GTGAGCTCGGTACCGGATCCGTCTCTCTGTAACTCTAGCC |
| TRV2-NbHLH11-F | AGGTTACCGAATTCTCTAGATGAGCTGAATTCGTTGA |
| TRV2-NbHLH1-R | GTGAGCTCGGTACCGGATCCTTATCAAACACAAGTTGCTT |
| 1305-NbERF1-F | AGTCCGGAGCTAGCTCTAGAATGAATTCAGCATGTAACC |
| 1305-NbERF1-R | CCCTTGCTCACCATGGATCCCAGTACATCTGGAGGTTCCAAC |
| AD-NbERF1-F | CCATGGAGGCCA1GTGAATTCATGAATTCAGCATGTAACC |
| AD-NbERF1-R | AGCTCGAGCTCGATGGATCCCAGTACATCTGGAGGTTCCAAC |
| 1305-NbbHLH1-F | GTCCGGAGCTAGCTCTAGAATGACTGATTACAGATTAC |
| 1305-NbbHLH1-R | CCCTTGCTCACCATGGATCCGCGTGTTTCAGCAACTCTG |
| AD-NbbHLH-F | CCATGGAGGCCAGTGAATTCATGACTGATTACAGATTAC |
| AD-NbbHLH-R | AGCTCGAGCTCGATGGATCCGCGTGTTTCAGCAACTCTG |
| TRV2-NbPMT-F | CTGTGAGTAAGGTTACCGAATTCGAAGCATTCTCACTTAAGATTG |
| TRV2-NbPMT-R | GTGAGCTCGGTACCGGATCCTGCTGGACCAATGGGATCAGA |
| TRV2-NbQPRT-F | CTGTGAGTAAGGTTACCGAATTCAAGGCTCGCAGCATTGTTAT |
| TRV2-NbQPRT-R | GTGAGCTCGGTACCGGATCCGTAACACCAGTTTGTCCAAT |
| pGreen-PMTpro-F | TCGACGGTATCGATAAGCTTAATAATGAAAAAGTAAAG |
| pGreen-PMTpro-R | GCTCTAGAACTAGTGGATCCTTTCAAGATTAAACTAAAGA |
| pBI121-PMTpro-F | GACCATGATTACGCCAAGCTTAAATGAAAAAGTAAAG |
| pBI121-PMTpro-R | GGACTGACCACCCGGGGATCCTTTCAAGATTAAACTAAAGA |
| pAbAi-PMTpro-F | TTGAATTCGAGCTCGGTACCAATAATGAAAAAGTAAAGAA |
| pAbAi-PMTpro-R | TACAGAGCACATGCCTCGAGTTTCAAGATTAAACTAAAGA |
| pGreen-QPRTpro-F | TCGACGGTATCGATAAGCTTCAGGGCTGCTCAGTAGAC |
| pGreen-QPRTpro-R | GCTCTAGAACTAGTGGATCCGGAAATTTTGGGGTGCGAAA |
| pBI121-QPRTpro-F | GACCATGATTACGCCAAGCTTCAGGGCTGCTCAGTAGAC |
| pBI121-QPRTpro-R | GGACTGACCACCCGGGGATCCGGAAATTTTGGGGTGCGAAA |
| pAbAi-QPRTpro-F | TTGAATTCGAGCTCGGTACCCAGGGCTGCTCAGTAGACA |
| pAbAi-QPRTpro-R | TACAGAGCACATGCCTCGAGGGAAATTTTGGGGTGCGAAA |
| pGreen-ERF1pro-F | TCGACGGTATCGATAAGCTTATTCTTTACTCAGTAGGA |
| pGreen-ERF1pro-R | GCTCTAGAACTAGTGGATCCTCCATAAACCCAGCACAAAA |
| pBI121-ERF1pro-F | AACACGGGGGACTCTAGAATTCTTTACTCAGTAGGA |
| pBI121-ERF1pro-R | GGACTGACCACCCGGGGATCCTCCATAAACCCAGCACAAAA |
| pAbAi-ERF1pro-F | TTGAATTCGAGCTCGGTACCATTCTTTACTCAGTAGGA |
| pAbAi-ERF1pro-R | TACAGAGCACATGCCTCGAGTCCATAAACCCAGCACAAAA |
| qNbActing-F | CAATCCAGACACTGTACTTTCTCTC |
| qNbActing-R | AAGCTGCAGGTATCCATGAGACTA |
| qGUS-F | ATCGCGAAAACTGTGGAATT |
| qGUS-R | TGCCCAACCTTTCGGTATAA |
